# Integrating Graphene Oxide and Mesenchymal Stem Cells in 3D-Printed Systems for Drug Delivery and Tissue Regeneration

**DOI:** 10.3390/pharmaceutics17081088

**Published:** 2025-08-21

**Authors:** Igor Soares Gianini Grecca, Vitor Fernando Bordin Miola, Júlia Carolina Ferreira, Thiago Rissato Vinholo, Laira Mireli Dias da Silva, Paulo Gabriel Friedrich Totti, Silvia Helena Soares Gianini, Maricelma da Silva Soares de Souza, Juliana da Silva Soares de Souza, Adriano Cressoni Araújo, Elen Landgraf Guiguer, Caio Sérgio Galina Spilla, Marcelo Dib Bechara, Domingos Donizeti Roque, Eliana de Souza Bastos Mazuqueli Pereira, Karina Torres Pomini

**Affiliations:** 1 School of Medicine, University of Marília (UNIMAR), Avenida Hygino Muzzy Filho, Marília 17525-902, SP, Brazil; 1939228@unimar.br (I.S.G.G.); vitorfbmiola@gmail.com (V.F.B.M.); 1941091@unimar.br (T.R.V.); 2 Department of Periodontics, School of Medicine, University of Marília (UNIMAR), Avenida Hygino Muzzy Filho, Marília 17525-902, SP, Brazil; juliaferrreira@unimar.br; 3 Interdisciplinary Pos Graduate Program in Structural and Functional Interactions in Rehabilitation, University of Marília (UNIMAR), Avenida Hygino Muzzy Filho, Marília 17525-902, SP, Brazil; lairadias@outlook.com.br (L.M.D.d.S.); 1897288@unimar.br (P.G.F.T.); araujo01@unimar.br (A.C.A.); eleng@unimar.br (E.L.G.); mbechara@unimar.br (M.D.B.); elianabastos@unimar.br (E.d.S.B.M.P.); 4 Department of Propaedeutics, School of Medicine, University of Marília (UNIMAR), Avenida Hygino Muzzy Filho, Marília 17525-902, SP, Brazil; silviagianini@unimar.br; 5 Department of Biochemistry and Pharmacology, School of Medicine, University of Marília (UNIMAR), Avenida Hygino Muzzy Filho, 1001, Marília 17525-902, SP, Brazil; maricelma.soares.souza@unimar.br; 6 Faculty of Veterinary Medicine, University of Marília (UNIMAR), Avenida Hygino Muzzy Filho, Marília 17525-902, SP, Brazil; 1994694@unimar.br; 7 Department of Human Morphophysiology, School of Medicine, University of Marília (UNIMAR), Avenida Hygino Muzzy Filho, 1001, Marília 17525-902, SP, Brazil; caiospilla@unimar.br (C.S.G.S.); dune.roque@unimar.br (D.D.R.); 8 Department of Prosthodontics, University of Marília (UNIMAR), Avenida Hygino Muzzy Filho, 1001, Marília 17525-902, SP, Brazil

**Keywords:** biocompatible material, cell differentiation, mesenchymal stem cell, nanomaterial, graphene oxide, regenerative medicine

## Abstract

Mesenchymal stem cells (MSCs) represent a promising strategy in the field of regenerative medicine due to their multipotent differentiation capacity and immunomodulatory properties. The interaction of these cells with the extracellular matrix (ECM) and biomaterials, notably graphene oxide (GO), has proven decisive in modulating cell behavior, with the potential to optimize tissue regeneration processes. This review was conducted using the MEDLINE, Scopus, and Cochrane databases, covering studies published between 2018 and 2025, from which seven studies met the inclusion criteria, with an emphasis on in vitro and in vivo investigations regarding the association between GO and MSCs. The main findings demonstrate that GO, particularly when conjugated with polymers such as poly(L-lactic acid) (PLLA), enhances cell adhesion, stimulates proliferation, and promotes the osteogenic differentiation of MSCs, in addition to positively modulating intracellular signaling pathways. However, significant gaps remain in understanding the mechanisms and safety of GO’s therapeutic use in association with MSCs. Therefore, this review reinforces the need for further studies to deepen the characterization of the bioactive properties of GO-MSCs, aiming to enable safer and more effective clinical applications.

## 1. Introduction

Mesenchymal stem cells (MSCs) have emerged as a promising source for regenerative therapies, intrinsically endowed with a remarkable capacity for proliferation, multipotent differentiation, and potent immunomodulatory action. Distributed across diverse human tissues, such as bone marrow, adipose tissue, dental pulp, endometrium, and skin, MSCs exhibit the ability to differentiate into multiple cell lineages, encompassing adipocytes, osteocytes, as well as neural and cardiac cells [1,2]. Furthermore, their expressive immunomodulatory effects qualify them as ideal candidates for the treatment of immunological and inflammatory conditions [3,4].

The notable versatility of MSCs has driven their successful application across a vast array of contexts within regenerative medicine. This spans from bone reconstruction and cartilage repair to musculoskeletal tissue regeneration, the restoration of components of the central and peripheral nervous systems, hepatic regeneration, and the reconstruction of structures such as the cornea, trachea, and skin. The efficacy of these applications, however, is intrinsically dependent on the creation of optimal microenvironments for their cultivation and effective differentiation [4,5,6].

In this context, the extracellular matrix (ECM) proves to be a fundamental element, exerting a critical regulatory role over MSC behavior. Its influence extends to vital cellular processes, including homeostasis, proliferation, regeneration, and differentiation [7]. The complex interplay between MSCs and ECM components can activate multiple signaling pathways, which intrinsically modulate cellular behavior and, consequently, the therapeutic outcome [8].

In response to the demand for controlled and structured environments, recent technological advancements in regenerative medicine have underscored the indispensability of biomaterials. These not only provide crucial structural support for MSCs but also modulate their biological responses. Among synthetic biomaterials—which include extensively investigated polymers, ceramics, and metals [9]—graphene oxide (GO) stands out as a particularly promising candidate. Its unique properties, such as biocompatibility, the remarkable capacity to promote cell adhesion, and high mechanical strength, position it prominently [5]. The chemical interaction between GO and MSCs, mediated by functional groups such as carboxyl, hydroxyl, and epoxide moieties, has been proven to optimize cell adhesion, proliferation, and differentiation [10,11].

The family of graphene materials, derived from graphite, encompasses various forms with distinct properties and applications, notably graphene (G), graphene oxide (GO), and reduced graphene oxide (rGO). Pure graphene (G) is a two-dimensional material with sp^2^ bonds and remarkable electrical and mechanical properties, characterized by its hydrophobicity and poor dispersibility in aqueous solvents, which limits its direct application in biological environments [12,13].

In contrast, graphene oxide (GO) is a chemically modified form of graphene extensively functionalized with oxygen-containing groups such as hydroxyls, carboxyls, and epoxides distributed throughout its structure. This abundance of functional groups confers upon GO high hydrophilicity and excellent dispersibility in water and biological solutions, a crucial characteristic for its integration into bioinks for 3D bioprinting and for interactions within aqueous biological systems [14,15]. Furthermore, the presence of these oxygen functionalities facilitates the chemical functionalization of GO, enabling the grafting of biomolecules, drugs, and polymers, which can modulate its biocompatibility and direct specific cellular interactions [15,16]. Although GO is typically insulating or semiconducting due to the disruption of its sp^2^ network, this property can be tuned, and its capacity to adsorb proteins and growth factors is fundamental to its bioactivity [11,17].

Reduced graphene oxide (rGO) represents an intermediate stage, where GO undergoes reduction processes to partially or fully remove oxygen groups. This reduction aims to restore some of the electronic properties of pristine graphene, making rGO more conductive than GO but generally less hydrophilic and dispersible than GO. Its properties are, therefore, a balance between G and GO, varying significantly with the degree of reduction [13].

In this review, the focus is placed on graphene oxide (GO) due to its unique combination of characteristics that render it particularly promising for the modulation of mesenchymal stem cells (MSCs) in 3D systems. GO’s superior hydrophilicity and dispersibility are essential for its incorporation into various bioinks and for the formation of 3D scaffolds compatible with cell culture. The abundance of oxygen functional groups on GO not only allows for easy surface modification to optimize biocompatibility and bioactivity but also facilitates specific interactions with biomolecules and cell adhesion, as discussed in depth in the subsequent sections. While rGO and G also demonstrate potential, GO’s surface properties and processability establish it as a highly versatile and relevant biomaterial for tissue engineering and regenerative medicine, justifying the centrality of its analysis in this review [16,18].

Despite the growing interest in the association between GO and MSCs, a significant gap persists in the literature concerning a comprehensive and up-to-date understanding of their integration, especially within 3D-printed systems for drug delivery and tissue regeneration. Existing studies, though valuable, frequently exhibit a restricted focus. For instance, Lasocka et al. predominantly addressed cutaneous applications with limited molecular evidence and restricted to topical dressings [19]; Zhou et al. demonstrated localized growth factor delivery via GO in 3D hydrogels, although circumscribed to cartilage and in vitro conditions [20]; and Sekuła-Stryjewska et al. (2021) performed advanced comparisons of GO sizes for cardiogenesis, albeit exclusively in vitro and without deeper mechanistic insights [21]. Similarly, the research by Ricci et al., while elucidating GO’s osteoinductive properties via BMP pathways, maintained a narrow focus on bone tissue and incredibly specific GO modifications, failing to provide a broader contextualization [22].

Additionally, preexisting reviews demonstrate considerable limitations. The comprehensive review by Maleki et al., for example, though seminal, did not incorporate crucial post-2020 advancements, such as recent GO functionalization, contemporary in vivo studies, or an in-depth analysis of safety and cytotoxicity challenges [23]. Analogously, a more recent overview generically addresses biocompatibility and “future trends” but lacks detailed practical cases and specific progress beyond 2021 [24]. Consequently, a critical and current appraisal that not only synthesizes these diverse applications but also delves into mechanistic complexities and evaluates the true translational potential of GO-MSC interactions in advanced 3D-printed systems, with particular emphasis on poly(L-lactic acid) (PLLA) nanocomposites, is urgently required. Overcoming inherent biocompatibility challenges and discerning the full scope of their utility remains essential for successful clinical translation.

Given the necessity for a comprehensive synthesis of evidence derived from diverse research designs, including in vitro and in vivo studies, this work has been structured as an integrative review. Through critical analysis of the impact of these polymeric composites on MSC cellular expression, this review aims to explore the properties of GO in association with MSCs, with particular attention given to existing knowledge gaps concerning their therapeutic use and anticipating their valuable contributions to future clinical applications.

## 2. Methodology

This integrative review was conducted while following a rigorous methodology to systematically identify, analyze, and synthesize the available scientific literature on the use of graphene oxide in conjunction with mesenchymal stem cells for regenerative medicine applications. The methodological steps adhered to established guidelines for integrative reviews, ensuring transparency and reproducibility. The illustrative figures and conceptual diagrams presented in this review were developed by the authors based on the synthesis of data and information from the cited literature.

### 2.1. Databases and Search Strategy

Searches were systematically performed in the MEDLINE-PubMed, Scopus, and Cochrane databases, covering studies published from 2018 to 2025. The search strategy was meticulously developed using a combination of controlled vocabulary (MeSH terms for PubMed) and keywords, employing Boolean operators (AND, OR) to ensure comprehensive yet precise coverage. The full search strings utilized for each database were as follows:

- MEDLINE-PubMed: ((“Mesenchymal Stem Cells” [Mesh] OR “Mesenchymal Stem Cells” [tiab]) AND (“Graphene Oxide” [Mesh] OR “Graphene Oxide” [tiab] OR “GO” [tiab]) AND (“Regenerative Medicine” [Mesh] OR “Regenerative Medicine” [tiab] OR “Tissue Engineering” [tiab])) AND (2018:2025 [dp]);
- SCOPUS: TITLE-ABS-KEY (“Mesenchymal Stem Cells”) AND TITLE-ABS-KEY (“Graphene Oxide” OR GO) AND TITLE-ABS-KEY (“Regenerative Medicine” OR “Tissue Engineering”);
- Cochrane Library: (Mesenchymal Stem Cells): ti,ab,kw AND (Graphene Oxide OR GO): ti,ab,kw AND (Regenerative Medicine OR Tissue Engineering): ti,ab,kw.

Additionally, to ensure a thorough and exhaustive search, reference lists of key articles and relevant reviews identified through the initial database searches were manually screened (snowballing) to identify any further pertinent studies not captured by the electronic search.

### 2.2. Eligibility Criteria

To ensure the relevance and scope of this review, primary studies published and written exclusively in English were considered. The inclusion criteria comprised clinical trials, investigative studies, retrospective studies, in vitro studies, and studies involving animal models. The exclusion criteria were as follows: articles not written in English, case reports, poster presentations, letters to the editor, as well as studies in which graphene oxide did not constitute the primary active material or the central focus of the investigation in combination with mesenchymal stem cells (MSCs). Additionally, studies specifically addressing ceramic nanocomposites, bactericidal activity assessments, oncogenic effect evaluations, the use of calcium-based culture media, or the employment of corticosteroids as the focus of the study were also excluded.

### 2.3. Study Selection

Articles identified through the search strategies in the PubMed (*n* = 148), Scopus (*n* = 157), and Cochrane Library (*n* = 12) databases were exported and subsequently imported into the Mendeley Desktop reference management software (Mendeley Reference Manager 2.105.0© 2023 Mendeley Ltd., all rights reserved). This step enabled the centralized organization of all search results.

For the identification and removal of duplicates, the integrated “Check for Duplicates” feature within Mendeley was employed. This feature automatically compares imported references based on metadata such as the title, authors, publication year, and digital object identifier (DOI).

The duplicate suggestions generated by the software were thoroughly reviewed and manually confirmed by the two independent reviewers (K.T.P. and I.S.G.G.). Following manual verification, the duplicated entries were merged into a single reference, ensuring that each study was evaluated only once. The total number of duplicates removed in this process will be recorded and presented in the review’s flow diagram.

After the deduplication process, the remaining unique articles underwent a rigorous two-phase screening process, which was conducted by two independent reviewers (K.T.P. and I.S.G.G.) to minimize bias.

In the first phase, the titles and abstracts of all unique articles were assessed against the predefined inclusion and exclusion criteria. Articles that did not clearly meet the eligibility criteria were excluded. Those deemed potentially relevant or about which there was any doubt or disagreement between the reviewers advanced to the next phase.

In the second phase, the full texts of the pre-selected articles were retrieved and meticulously evaluated by both independent reviewers against all eligibility criteria. Any discrepancies or disagreements regarding the inclusion or exclusion of an article were resolved through discussion and consensus between the reviewers. In the event of an impasse, a third reviewer (V.F.B.M.) would be consulted to mediate the final decision.

**Figure 1:**
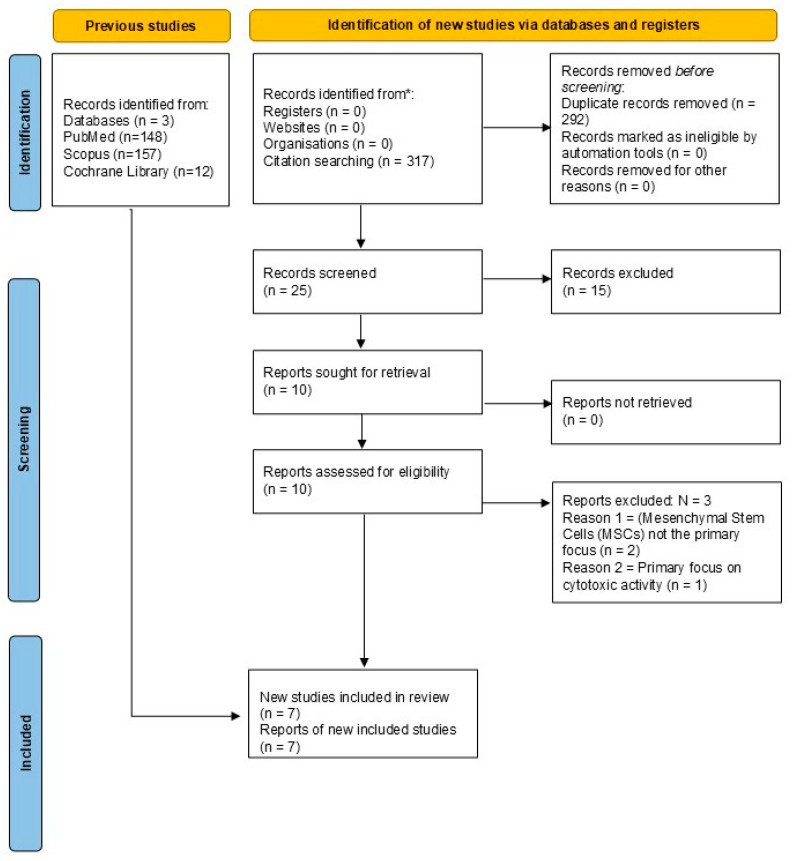
PRISMA 2020 flow diagram for new systematic reviews, which includes searches of databases and registers.

The number of articles identified at each selection phase and the reasons for exclusion will be detailed in a flow diagram (adapted from Preferred Reporting Items for Systematic Reviews and Meta-Analyses (PRISMA)), as presented in Figure 1 [24].

### 2.4. Data Collection

Data extraction from the 7 included studies was conducted using a standardized data extraction form developed by the authors and implemented in an electronic spreadsheet in Microsoft Excel^®^. This instrument was specifically designed to capture relevant information from each study, thereby ensuring data consistency and comprehensiveness. The extracted data included but were not limited to the principal author(s), publication year, country of study origin, study type (e.g., in vitro or in vivo), experimental model employed, details regarding the composition and concentration of graphene oxide (GO), type and source of mesenchymal stem cells (MSCs), co-culture or application methodology, outcomes assessed related to tissue regeneration, and main findings or results.

To ensure rigorous and unbiased data collection, two reviewers (D.D.R. and C.S.G.S.) independently extracted the data. The data extracted by each reviewer were subsequently compared. Any discrepancies identified were discussed and resolved by consensus between the two reviewers. In the absence of consensus, a third reviewer (M.D.B.) would be consulted to make the final decision, thereby ensuring data accuracy.

## 3. Literature Review

### 3.1. Properties of Mesenchymal Stem Cells (MSCs)

Mesenchymal stem cells (MSCs) are multipotent cells found in various human tissues, including bone marrow, adipose tissue, dental pulp, endometrium, and skin. They possess the capacity to differentiate into multiple cell lineages, such as adipocytes, osteocytes, neural cells, and cardiac cells. Additionally, MSCs exhibit immunomodulatory properties, making them suitable for the treatment of immunological and inflammatory conditions [4]. MSCs can be identified by specific surface markers, such as CD105 (endoglin), CD73 (5′-nucleotidase ecto), and CD90 (Thy-1), and are negative for hematopoietic markers like CD45 (protein tyrosine phosphatase, receptor type C), CD34 (hematopoietic stem cell glycoprotein), CD14 (monocyte and macrophage differentiation antigen), CD11b (integrin alpha M), CD79a (B-cell antigen receptor complex alpha chain), CD19 (B-cell differentiation antigen), and HLA-DR (human leukocyte antigen-DR) [25].

#### 3.1.1. Differentiation

MSCs can differentiate into various cell types, including chondrogenic (cartilage), osteogenic (bone), and adipogenic (fat) lineages, which are fundamental for tissue repair and regeneration. This differentiation potential is influenced by a range of stimuli, including cytokines, growth factors, and the features of the local microenvironment [6].

#### 3.1.2. Immunomodulation

MSCs modulate the immune response by reducing inflammation and preventing transplant rejection. They secrete a range of bioactive molecules that influence immune cell activity and contribute to an anti-inflammatory environment. These properties make MSCs promising candidates for therapies targeting autoimmune and inflammatory diseases [26].

#### 3.1.3. Angiogenesis

MSCs promote angiogenesis, i.e., the formation of new blood vessels, by secreting growth factors and other signaling molecules. This is essential for wound healing and tissue regeneration [27].

#### 3.1.4. Secretome

The MSC secretome, comprising diverse proteins, peptides, and RNAs, plays a key role in modulating the tissue microenvironment and enhancing regenerative processes. The secretome includes factors that promote cell survival, proliferation, and differentiation [28].

#### 3.1.5. Tissue Regeneration and Repair

Thanks to their differentiation potential and secretion of growth factors, MSCs significantly contribute to the regeneration of damaged tissues, such as bone, cartilage, and skeletal muscle. Their ability to interact with the local microenvironment and other cell types enhances their regenerative capabilities [29].

#### 3.1.6. Interaction with Stem Cells and Niches

MSCs interact with other stem cell types and the cellular niches where they reside. These interactions influence both MSC behavior and the function of other resident cells in the microenvironment. The MSC niche, i.e., their specialized microenvironment, dynamically regulates their behavior through interactions with the extracellular matrix and surrounding cells [4].

#### 3.1.7. Biological Functions and Cell Signaling of MSCs

MSCs play a fundamental role in tissue homeostasis and regeneration. Their key biological functions include proliferation, multipotency, homing and migration, trophic support, and immunosuppression. These cells possess a remarkable self-renewal capacity, ensuring a constant pool of undifferentiated cells for regenerative purposes. Cell signaling is central to MSC function. Intracellular and intercellular signaling pathways such as Wnt, Notch, and TGF-β regulate self-renewal, lineage commitment, and responses to external stimuli. Understanding these mechanisms is crucial for effectively manipulating MSCs in therapeutic applications [30].

Cell signaling is central to MSC function. Intracellular and intercellular signaling pathways such as Wnt, Notch, and TGF-β regulate self-renewal, lineage commitment, and responses to external stimuli. Understanding these mechanisms is crucial for effectively manipulating MSCs in therapeutic applications [31].

MSCs, also referred to as stromal stem cells, are valuable due to their intrinsic ability to differentiate into diverse cell types. Derived from tissues such as bone marrow, adipose tissue, and the umbilical cord, MSCs are central to regenerative therapies due to their adaptability and functional versatility. The differentiation process entails significant morphological and molecular changes. When directed toward specific lineages (e.g., osteogenic, chondrogenic, or adipogenic), MSCs undergo tightly regulated transformations mediated by signaling pathways and differential gene expression. This process is influenced by a range of stimuli, including cytokines, growth factors, and the features of the local microenvironment. The ability to modulate these cues under in vitro conditions provides a valuable tool for controlling and directing MSC differentiation [31].

#### 3.1.8. Growth Factors, Cytokines, and Biologically Active Molecules

##### Growth Factors

- Platelet-derived growth factor (PDGF) stimulates cell proliferation and migration, playing a key role in wound healing and tissue regeneration [32].
- Vascular endothelial growth factor (VEGF) promotes angiogenesis—the formation of new blood vessels—which is essential for tissue repair and regeneration [33].
- Transforming growth factor beta (TGF-β) regulates cell proliferation, differentiation, and extracellular matrix production. It plays a crucial role in fibrogenesis and modulation of inflammatory processes [34].
- Epidermal growth factor (EGF) stimulates cell proliferation and differentiation and participates in tissue repair and wound healing [35].
- Insulin-like growth factor 1 (IGF-1) supports cell survival, growth, and differentiation and contributes to bone and cartilage regeneration [36].
- Fibroblast growth factor (FGF) is involved in tissue regeneration and angiogenesis by promoting cell proliferation and differentiation [37].
- Bone morphogenetic protein 2 (BMP-2) plays a fundamental role in cell differentiation and tissue remodeling. Produced by chondrocytes, it promotes cartilage and bone formation and has emerged as a promising agent for cartilage repair and chondrogenesis. However, its anabolic activity may also lead to aggrecan degradation and cartilage alterations, reflecting the complex nature of its cellular effects. Studies have shown that intra-articular administration of BMP-2 can induce osteophyte formation, indicating both regenerative and adverse effects [38]. BMP-2 interacts with matrix-degrading enzymes, influencing extracellular matrix homeostasis. A comprehensive understanding of BMP-2-mediated intracellular signaling is essential to harness its therapeutic potential while minimizing adverse outcomes [39].
- Bone morphogenetic protein 4 (BMP-4), a member of the TGF-β superfamily, was initially identified in bone extracts. It shows therapeutic potential in treating long bone fractures, osteoarthritis, rheumatoid arthritis, and cartilage regeneration. In vitro, BMP-4 supports the self-renewal of mouse embryonic stem cells (ESCs) when combined with leukemia inhibitory factor (LIF). Additionally, it has been used as a serum substitute for the expansion of human ES and iPS cells. Nevertheless, its application requires precise dosing and ethical considerations, highlighting the need for careful clinical use [40].
- Bone morphogenetic protein 7 (BMP-7) is another key member of the TGF-β family and is crucial for MSC differentiation. It is known to induce osteogenesis and promote bone tissue formation. It also contributes to maintaining MSC pluripotency and is involved in adipogenesis. During embryonic development, BMP-7 plays a central role in tissue and organ formation. Beyond its classical functions, BMP-7 modulates inflammatory responses and participates in tissue repair, making it a candidate for therapeutic strategies. However, its effects depend heavily on the cellular microenvironment and culture conditions [41].
- FGF-2 and VEGF: Fibroblast growth factor 2 (FGF-2) and vascular endothelial growth factor (VEGF) are both involved in regulating MSC differentiation. FGF-2, also known as basic FGF (bFGF), promotes proliferation and can guide MSCs toward specific lineages. VEGF is primarily associated with angiogenesis but also plays a role in MSC fate determination. Its expression varies depending on external stimuli and pathological conditions. The synergistic interaction of FGF-2, VEGF, and environmental factors critically influence MSC behavior in various tissues [42].

##### Cytokines

- Interleukin-6 (IL-6) is a pleiotropic cytokine that regulates inflammation and modulates immune response. It can act as either pro-inflammatory or anti-inflammatory, depending on the context [43].
- Interleukin-10 (IL-10) is an anti-inflammatory cytokine that plays a central role in immune regulation, supporting the balance between inflammation and regeneration [44].
- Tumor necrosis factor alpha (TNF-α) is a key regulator of inflammation with both pro-inflammatory and regenerative properties, depending on the tissue context [45].

##### Biologically Active Molecules

- Prostaglandins are lipid-derived signaling molecules synthesized from arachidonic acid which are involved in inflammation modulation and injury response [46].
- Matrix metalloproteinases (MMPs) is a family of enzymes that degrade extracellular matrix components, facilitating tissue remodeling and wound healing [47].
- Soluble cytokines include chemokines, which recruit inflammatory cells to injury sites and orchestrate local immune responses [48].
- Exosomes are nano-sized extracellular vesicles secreted by MSCs that contain proteins, lipids, and RNAs. They mediate intercellular communication and influence immune and inflammatory responses [49].
- Wnt-3, a canonical Wnt signaling pathway activator, is highly expressed in the dorsal midline region and is essential for spinal cord development. During vertebrate embryogenesis, it regulates self-renewal, proliferation, differentiation, and cell motility. Its activation governs cell fate decisions and plays a pivotal role in directing MSC differentiation into specific lineages [50].
- Wnt-4 is one of the most studied ligands of the Wnt family, which comprises 19 genes involved in stem cell regulation, development, and oncogenesis. Wnt-4 plays a key role in organogenesis, including sex determination and mammary gland development. Its dysregulation is linked to reduced bone mineral density, premature skeletal aging, endometriosis, and gynecological cancers. Wnt-4 can activate both β-catenin-dependent and independent pathways, acting as either an activator or suppressor, depending on the context. This functional duality highlights the need for further investigation into its role in MSC differentiation [51].
- Transforming growth factor beta (TGF-β) is ubiquitously expressed and essential for normal development and tissue homeostasis. There are three isoforms—TGF-β1, TGF-β2, and TGF-β3—which signal via binding to TGFBR2, triggering phosphorylation of TGFBR1. This activates SMAD2/3, which complexes with SMAD4 and translocates to the nucleus to regulate gene expression. This canonical signaling cascade is fundamental to controlling proliferation, differentiation, migration, and apoptosis. TGF-β plays an essential role in embryonic development and in maintaining adult tissue equilibrium [52].
- β-catenin is the central mediator of the canonical Wnt/β-catenin signaling pathway, and its cytosolic concentration is tightly regulated. In the absence of Wnt stimulation, cytosolic β-catenin is actively phosphorylated by a destruction complex comprising adenomatous polyposis coli (APC), Axin, glycogen synthase kinase 3β (GSK3β), casein kinase 1α (CK1α), and protein phosphatase 2A (PP2A). Within this complex, APC and Axin function as scaffolding proteins, facilitating β-catenin phosphorylation at Ser45 by CK1α and at Ser33, Ser37, and Thr41 by GSK3β. These phosphorylation events trigger ubiquitination and proteasomal degradation of β-catenin. Consequently, under basal conditions, β-catenin levels are maintained at a minimum. Upon Wnt stimulation, this phosphorylation cascade is disrupted, preventing β-catenin degradation. As a result, β-catenin accumulates in the cytosol and subsequently translocates into the nucleus, where it interacts with transcription factors to activate the expression of target genes involved in cell proliferation, differentiation, and survival. This tightly regulated mechanism underscores the pivotal role of β-catenin in mesenchymal stem cell (MSC) fate determination, highlighting its importance in orchestrating molecular pathways central to stem cell biology [53].

##### Wnt/β-Catenin Pathway

The Wnt/β-catenin signaling pathway consists of four key segments: extracellular, membrane, cytoplasmic, and nuclear. The extracellular segment involves Wnt ligands, such as Wnt3a, Wnt1, and Wnt5a. The membrane segment includes the primary receptors Frizzled and LRP5/6. The cytoplasmic segment comprises core signaling components including β-catenin, Dishevelled (DVL), GSK-3β, Axin, APC, and CK1. In the nucleus, β-catenin binds to T-cell factor/lymphoid enhancer factor (TCF/LEF) transcription factors, thereby modulating the transcription of key target genes, such as c-Myc and matrix metalloproteinases (MMPs). This signaling cascade plays a fundamental role in the regulation of essential biological processes and is critical for cell fate determination, especially in stem cell differentiation contexts. The pathway’s complexity reflects its significance in both developmental biology and regenerative medicine [54].

Table 1 provides a comprehensive summary of the key growth factors, cytokines, and biologically active molecules discussed in this section. This table systematically organizes the information, highlighting the functions and references associated with each molecule and thereby facilitating a clearer understanding of their roles in mesenchymal stem cell biology and regenerative medicine.

### 3.2. Graphene Oxide (GO)-Based Nanomaterials

Graphene oxide (GO)-based nanomaterials have demonstrated significant potential in regenerative therapies, particularly when combined with polymers such as poly(L-lactic acid) (PLLA). The following sections outline the key properties and advantages of this combination.

#### 3.2.1. Properties of Graphene Oxide

GO exhibits excellent electrical conductivity, which is beneficial in applications involving electrical stimulation of cells. This property is particularly relevant in promoting cell differentiation, especially in neural and stem cell-based therapies [57].

GO possesses an exceptionally large surface area (>2600 m^2^/g), enabling enhanced interaction with cells and biomolecules. This facilitates the adsorption of bioactive agents and improves the efficacy of controlled drug delivery systems, while also promoting cell adhesion through optimized cell–matrix interactions.

GO demonstrates satisfactory biocompatibility, especially when functionalized with chemical groups that reduce intrinsic toxicity. This enables its safe use in biological systems, supporting high cell viability in experimental models [58].

#### 3.2.2. Advantages of Poly(L-Lactic Acid) (PLLA)

PLLA is a biodegradable polymer widely used in regenerative medicine due to its controlled degradation rate, which releases byproducts at levels compatible with tissue repair and remodeling [59,60,61]

PLLA displays adequate mechanical strength for supporting tissue regeneration and can withstand physiological loads. It also offers flexibility for shaping into various scaffold architectures [61].

PLLA can be physically and chemically modified to adjust properties such as the porosity, hydrophilicity, and degradation rate. Techniques such as copolymerization and nanoparticle functionalization enhance their versatility and application scope [59].

#### 3.2.3. PLLA/GO Combination

##### Enhanced Scaffolds

The integration of GO into PLLA matrices enhances both the mechanical strength and electrical conductivity of the resulting scaffolds. These hybrid materials promote cell adhesion, proliferation, and osteogenic differentiation, making them highly promising for bone tissue engineering [12,59,62].

##### Scaffold Fabrication Methodologies for GO/PLLA Composites

Tissue engineering with graphene oxide (GO) and poly(L-lactic acid) (PLLA) composites necessitates the careful selection of scaffold fabrication methodologies that can translate the inherent properties of these materials into three-dimensional structures with optimal characteristics for mesenchymal stem cell (MSC) support and modulation. The choice of fabrication technique critically impacts the scaffold’s architecture (porosity, pore size and interconnectivity, and surface area), its mechanical properties, and the ability to incorporate and control the distribution of biomolecules, in addition to influencing cell viability and behavior. Various methods have been employed, each with its specific advantages and disadvantages concerning GO/PLLA (Table 2).

While 3D printing, particularly extrusion (fused deposition modeling (FDM)) and light-based techniques (stereolithography (SLA) and digital light processing (DLP)), offers unprecedented control over scaffold architecture, enabling the creation of complex, customized structures with micrometric precision, the presence of GO in bioinks can introduce challenges in optimizing viscosity and preventing aggregation. Electrospinning, on the other hand, excels at mimicking the nanofibrous structure of the ECM, but creating thick 3D scaffolds with a controlled porosity throughout their depth remains a challenge. Techniques such as particulate leaching and freeze-drying are simpler and more economical but offer less control over the final microarchitecture than 3D printing. The choice of the ideal method for GO/PLLA scaffolds, therefore, critically depends on the specific biomedical application and the requirements for tissue mimicry.

##### Drug Delivery

GO serves as an efficient drug delivery platform, enabling the encapsulation and controlled release of bioactive compounds. The incorporation of GO into PLLA scaffolds improves the therapeutic efficacy of composite systems [55,56,57]. In the context of drug delivery, a field of increasing interest in biomaterials, graphene oxide (GO) and poly(L-lactic acid) (PLLA) composites emerge as promising platforms. Their combined properties, such as GO’s high surface area for drug adsorption and PLLA’s controlled biodegradability, offer versatility in developing delivery systems. However, to optimize their effectiveness, it is fundamental to understand the inherent advantages and disadvantages associated with their use of different delivery strategies. Table 3 presents the main advantages of GO/PLLA composites for drug delivery efficiency, while Table 4 details the disadvantages and challenges.

However, despite these advantages, the application of GO/PLLA composites also presents several inherent disadvantages and challenges that need to be addressed, as detailed in Table 4.

As Table 3 and Table 4 illustrate, the use of graphene oxide (GO)/poly(L-lactic acid) (PLLA) composites for drug delivery presents a duality. GO’s high surface area and diverse functional groups enable interactions with a wide range of pharmacological molecules, allowing for high drug loading [73]. This, coupled with PLLA’s polymeric structure allowing for prolonged and controlled release, gives these composites valuable flexibility for designing delivery systems with customized release profiles [74]. The possibility of targeted functionalization of GO minimizes systemic toxicity and maximizes the therapeutic effect at specific sites, such as tumors or inflamed areas, by enabling precise delivery [75].

Despite their significant potential, challenges persist. Nonspecific drug adsorption onto GO, particularly in complex biological fluids, can affect the intended release kinetics and lead to unintended interactions [76]. Furthermore, variability in GO synthesis and processing can impact the material’s properties and, consequently, its performance, demanding more sophisticated surface functionalization approaches and stringent quality control to ensure uniformity and clinical efficacy [77]. PLLA’s degradation rate, while offering biodegradability, can sometimes be too slow for specific applications or may generate acidic byproducts that need careful consideration [78]. Additionally, the potential for an initial “burst release” of the loaded drug, which is often undesirable for sustained therapeutic effects, requires careful optimization of the composite’s architecture and drug-material interactions to achieve the desired controlled release profile and prevent adverse effects [79].

Future research in this promising field should focus on integrating multi-stimuli responses into GO/PLLA systems for truly on-demand drug release, allowing for precise control triggered by physiological changes or external cues [80]. Moreover, standardizing production methods is critical to ensure batch-to-batch reproducibility and scalability for potential clinical translation, thereby solidifying the use of GO/PLLA composites as promising and reliable drug carriers.

##### Cellular Stimulation

The conductive properties of GO, in conjunction with PLLA’s structural support, provide an optimal environment for electrical stimulation of cells. This feature is particularly beneficial for stem cell differentiation into specialized lineages, such as osteogenic or myogenic pathways [62].

### 3.3. Applications for Regenerative Therapy

**Figure 2:**
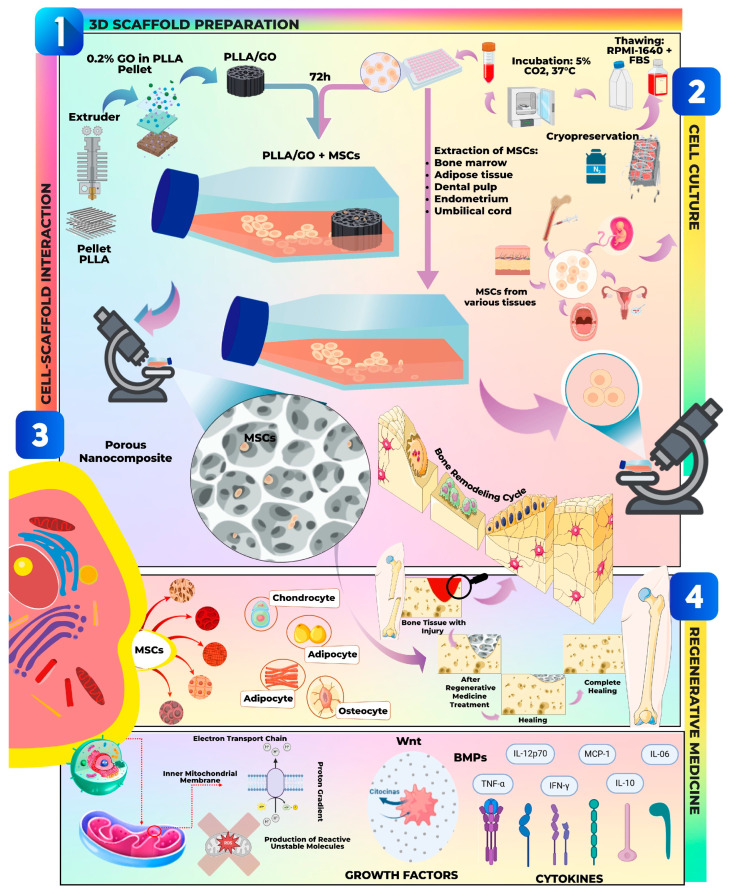
Schematic representation of the tissue engineering process using mesenchymal stem cells (MSCs) and poly(L-lactic acid) (PLLA) scaffolds functionalized with graphene oxide (GO) applications in regenerative medicine. (1) Preparation of the 3D scaffold. PLLA is processed into pellets and combined with 0.2% GO, forming a porous 3D scaffold through extrusion. The incorporation of GO enhances mechanical properties and biocompatibility, promoting MSC adhesion and proliferation. (2) MSC cell culture. MSCs are isolated from various sources and cryopreserved. After thawing, they are incubated in RPMI-1640 medium supplemented with FBS, ensuring cell viability and expansion. (3) Cell-scaffold interaction. MSCs are seeded onto the porous PLLA/GO scaffold. Micrography shows MSC interaction with the matrix, facilitating nutrient exchange and new tissue formation. (4) Regenerative medicine. MSCs differentiate into chondrocytes, adipocytes, and osteocytes, which are essential for tissue regeneration. The bone remodeling cycle illustrates the regeneration of injured bone tissue, with growth factors and cytokines modulating the inflammatory response and promoting tissue regeneration.

Within the context of regenerative medicine, the intricate process of tissue engineering leveraging graphene oxide (GO) functionalized poly(L-lactic acid) (PLLA) scaffolds in conjunction with mesenchymal stem cells (MSCs) is crucial for various therapeutic applications. Figure 2 provides a schematic representation detailing the sequential stages of this tissue engineering approach, from scaffold preparation and cell culture to the pivotal cell-scaffold interaction and the ultimate applications in regenerative medicine.

#### 3.3.1. Bone Regeneration

PLLA/GO scaffolds have been extensively studied for bone tissue engineering. These composites promote extracellular matrix mineralization and upregulate osteogenic gene expression in MSCs, providing both mechanical support and biochemical cues for osteogenesis [24,81,82].

#### 3.3.2. Soft Tissue Regeneration

GO-based nanocomposites also show promise in soft tissue regeneration, where cellular interaction and electrical conductivity are essential. PLLA/GO scaffolds facilitate cell communication and functional regeneration of tissues such as skin and peripheral nerves [82].

#### 3.3.3. Combination Therapies

The functionalization of GO allows the incorporation of growth factors, bioactive proteins, or stem cells, enabling combination therapies that accelerate and enhance regenerative outcomes. This approach can significantly improve the effectiveness of tissue repair strategies [81].

### 3.4. Properties and Production of Graphene Oxide

Nanomaterials are unique substances whose dimensions fall between those of molecules and submicrometric particles. They exhibit properties remarkably distinct from those of molecular compounds and traditional crystalline solids. Their versatility, biocompatibility, and biostability arise from surface modifications and functionalization. Nanomaterials are typically classified into two major categories: inorganic nanomaterials (e.g., metals, metal hydroxides, and metal oxides) and organic nanomaterials, which include carbon nanotubes, graphene, and graphene oxide (GO) [11,81,83].

Among these, graphene oxide (GO) has emerged as a highly promising organic nanomaterial across diverse applications, notably in the differentiation of mesenchymal stem cells (MSCs). GO exhibits a high specific surface area, a rich abundance of oxygen-containing functional groups, and excellent mechanical strength, thermal stability, and electrical conductivity [83]. The synthesis of GO involves exfoliating graphite and oxidizing graphene [16].

Graphite consists of multiple stacked layers of sp^2^-hybridized carbon atoms, also known as graphene sheets, held together by van der Waals forces, which are weak electrostatic interactions responsible for the cohesion between layers [16]. The purpose of exfoliation is to disrupt these interlayer forces, thereby isolating individual graphene entities. Graphite exists in both natural and synthetic forms. Natural graphite is found to be amorphous, crystalline, or flake graphite, while synthetic graphite is produced by subjecting non-graphitic carbon materials, such as coke, to elevated temperatures under inert atmospheres or vacuum conditions [82].

Several methods have been developed to synthesize graphene and its derivatives, including microexfoliation, which mechanically peels graphite layers using adhesive tapes; chemical vapor deposition (CVD), involving graphene growth on metal substrates; and the widely adopted Hummers’ method, considered the most efficient and scalable technique [61]. The development of GO production methodologies has a long history. The earliest documented attempt dates to 1859, when B.C. Brodie reacted graphite with potassium chlorate (KClO_3_) in concentrated nitric acid (HNO_3_). The resulting compound, composed of carbon, hydrogen, and oxygen, was named “graphon”, and further oxidative treatments increased the oxygen content, which stabilized after four iterations. Upon heating, the compound released carbonic acid and carbon monoxide, suggesting successful oxidation of the graphite structure [16].

In 1898, Staudenmaier refined Brodie’s approach by gradually adding potassium chlorate during the reaction with nitric acid, thereby enhancing the degree of oxidation. Finally, the Hummers’ method, introduced by Hummers and Offeman in 1958, revolutionized GO production. This method utilizes a combination of potassium permanganate (KMnO_4_) and concentrated sulfuric acid (H_2_SO_4_) to oxidize graphite. The process involves acid-mediated oxidation followed by reduction, which can be either chemical or thermal, to yield GO with desirable surface functionalities and structures for various biomedical and engineering applications [84].

Graphene oxide, an amphiphilic and water-miscible material, contains a wide range of functional groups—such as oxygen, ketone, carboxyl, hydroxyl, and epoxy—which can be distributed both on the basal planes and at the sheet edges. These functional groups facilitate the combination of GO with other materials. The high oxygen content increases its hydrophilicity and biocompatibility while also preventing agglomeration in cell culture media [1]. This graphene derivative exhibits versatile functionality across numerous application fields, notably influencing the self-renewal and differentiation of mesenchymal stem cells. Its capacity to interact with growth factors during stem cell differentiation makes it a highly effective carrier in regenerative contexts. In bone tissue engineering, for instance, GO exerts a protective role, contributing significantly to vivo bone regeneration processes [83].

To assess the physicochemical characteristics of GO, several analytical techniques are employed. UV-Vis and fluorescence spectroscopy are used to evaluate optical properties, while Raman spectroscopy and zeta potential measurements provide insights into the surface characteristics. Thermogravimetric analysis (TGA) further allows the evaluation of the degree of functionalization of GO flakes [11].

A critical property of graphene oxide in biomedical applications is biocompatibility. Biocompatibility refers to the ability of a material to interact with biological tissues without eliciting adverse reactions, in contrast to toxicity, which is defined by harmful biological responses. Factors such as the particle size, shape, structure, and surface functionalization significantly influence biocompatibility outcomes [1]. GO’s unique structural characteristics—including the planar arrangement of sp^2^-hybridized carbon atoms, its high specific surface area, and the presence of oxygen-containing groups—confer it with excellent properties in terms of biocompatibility, physiological solubility, chemical stability, and drug delivery potential [16]. Compared with pristine graphene, GO exhibits notable differences, such as the absence of visible light absorption and greater chemical reactivity. Importantly, the surface of GO can be functionally modified, allowing tailored applications in biomedicine. Capitalizing on these attributes, the field of tissue engineering has advanced the development of three-dimensional structures known as scaffolds.

These 3D frameworks are designed to host and spatially organize cells, providing support for tissue development. Their porous architecture is especially beneficial for cell adhesion and proliferation and the promotion of differentiation. Additionally, scaffolds must possess biologically appropriate features such as adequate porosity, mechanical strength, and biocompatibility to effectively integrate into host tissues [80].

Among GO’s most relevant chemical transformations is its reduction to forms that approximate the properties of pristine graphene. Three principal reduction techniques are used to achieve this. Chemical reduction typically involves the dispersion of GO in colloidal suspension, followed by treatment with reducing agents such as hydrazine monohydrate. Thermal reduction, on the other hand, relies on heating GO in a furnace to achieve exfoliation and reduction, yielding thermodynamically stable carbon oxide species without the use of chemical agents.

Finally, electrochemical reduction offers a greener alternative by reducing oxygen-containing groups via electrochemical methods, thus avoiding hazardous reagents and minimizing byproduct formation [12].

A widely debated aspect in the context of graphene oxide concerns its cytotoxicity and the potential systemic effects associated with its use. The central issue raised by numerous researchers is the absence of a clear consensus regarding the possible adverse outcomes stemming from the administration of graphene oxide, particularly in human subjects [84]. Furthermore, systemic effects are influenced by multiple variables, including the particle size, concentration, exposure duration, and route of administration [61,71]. However, studies have shown that functionalization with polyethylene glycol (PEG) can mitigate cytotoxicity, improve aqueous stability, enhance biocompatibility, and increase the proliferation rates of mesenchymal stem cells. These enhancements suggest that PEGylated graphene oxide may offer superior advantages compared with the unmodified material.

### 3.5. Cytotoxic Activity of Graphene Oxide-Based Nanomaterials

The cytotoxic activity of graphene oxide (GO)-based nanomaterials is a critical area of investigation, particularly considering their potential biomedical and environmental applications. Cytotoxicity may vary significantly depending on the specific characteristics of the nanomaterial, including the particle size, shape, degree of oxidation, surface functionalization, and administered dose [61]. Below is a summary of the primary factors influencing the cytotoxicity of these materials (Table 2).

#### 3.5.1. Factors Influencing Cytotoxicity

##### Particle Size and Shape

The size of nanoparticles significantly influences their cytotoxic potential. Smaller particles present a larger surface-area-to-volume ratio, increasing the likelihood of interactions with biomolecules and cellular components. Additionally, smaller dimensions facilitate membrane penetration and tissue infiltration, which may enhance harmful effects, including disruption of cell integrity and the induction of programmed cell death [82].

The morphology of nanoparticles also plays an important role. Spherical, lamellar, or elongated shapes interact differently with cells, affecting endocytosis and membrane dynamics. Lamellar-shaped particles, characteristic of graphene oxide, are particularly associated with mechanical disruption of the cell membrane [16].

##### Degree of Oxidation and Functionalization

GO is rich in oxygen-containing functional groups such as epoxides, hydroxyls, and carboxyls. These groups can interact with cellular membranes and intracellular proteins, potentially triggering oxidative stress and metabolic disturbances. The high oxidation state of GO also increases its hydrophilicity, enhancing its dispersion in aqueous media and affecting its biodistribution [59].

The reduction of GO decreases the quantity of oxygenated functional groups, improving the electronic properties of the material. However, this reduction may also lower its cytotoxic potential by limiting interactions with cellular structures, resulting in reduced toxicity compared with GO [62].

##### Dose and Concentration

The toxic effects of GO and rGO are dose-dependent. Higher concentrations are frequently associated with increased cytotoxicity, manifesting as elevated oxidative stress, inflammatory responses, and cell death via necrosis or apoptosis [51].

##### Exposure Time

The duration of exposure to nanomaterials is a key determinant of toxicity. Prolonged exposure increases the probability of intracellular accumulation and cumulative damage, intensifying toxic outcomes [53].

##### Cell Type

**Figure 3:**
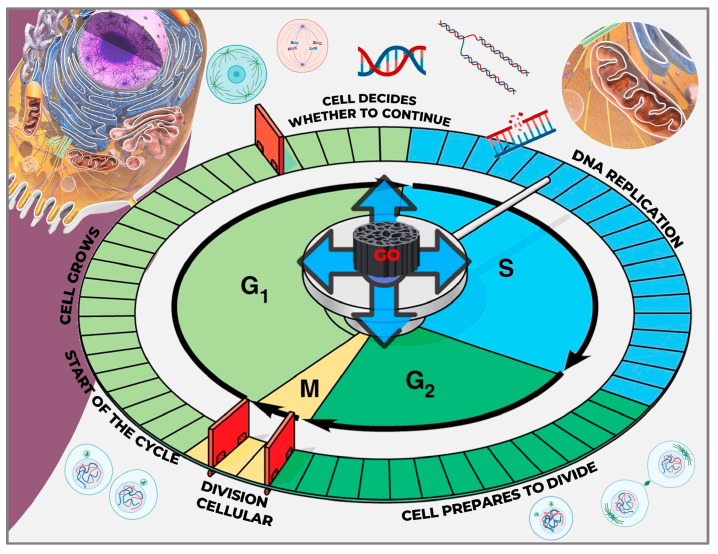
The cell cycle illustrates the sequence of cell growth and division (G1, S, G2, and M) and key regulatory checkpoints. Graphene oxide cytotoxicity is cell type-dependent, reflecting variations in metabolic activity, cell cycle phase, and the expression of specific cell surface receptors.

The cytotoxicity of GO varies across different cell types. Cells differ in metabolic activity, cell cycle phase, and expression of specific receptors, all of which can influence their susceptibility to nanomaterials and the extent of cytotoxic effects observed (Figure 3) [71].

##### Cytotoxic Mechanisms

One of the primary mechanisms of GO-induced toxicity is the generation of reactive oxygen species (ROS), which can oxidize membrane lipids, proteins, and nucleic acids, leading to substantial cellular damage (Table 5) [54].

The presence of oxygen-containing functional groups in GO can activate inflammatory pathways, stimulating the production of pro-inflammatory cytokines such as TNF-α and IL-6. This inflammatory cascade may disrupt tissue homeostasis [54,85].

Direct contact between GO and the cell membrane may compromise membrane integrity, alter permeability, and induce cell death [33,86].

GO nanoparticles internalized via endocytosis can accumulate within intracellular compartments such as lysosomes, causing localized toxicity and interfering with essential cellular functions [86].

##### Studies and Examples

Cell culture models are frequently used to assess the cytotoxicity of GO and rGO. Assays such as MTT, ROS quantification, and the evaluation of oxidative stress markers provide valuable insight into nanomaterial toxicity. One study investigated the interaction between GO and rGO with polymeric dispersants, including glycol chitosan, propylene glycol alginate (PGA), and polydopamine, and evaluated their effects on human chondrocytes using lactate dehydrogenase (LDH) assays. Additionally, the effects of fullerenes, another carbon-based nanomaterial, on intracellular ROS regulation and antioxidant capacity in fetal lung fibroblasts were assessed. The results indicated that GO’s effects are dependent on PGA’s presence and concentration. Moreover, exposure to water-soluble glycofullerene showed no adverse cellular effects, supporting its potential for drug delivery applications in pancreatic cancer treatment [59].

Animal models allow for evaluation of the systemic effects and biodistribution of nanomaterials, contributing to risk assessment in clinical and environmental contexts. In vitro studies have reported nanotoxic effects on human primary cells, demonstrating that titanium dioxide (TiO_2_), zinc oxide (ZnO), and cerium dioxide (CeO_2_) nanoparticles can induce both primary and oxidative DNA damage in salivary leukocytes. Although ZnO did not result in immune alterations, CeO_2_ showed low toxicity in acute exposure. However, long-term exposure increased the expression of apoptotic markers such as p53 and autophagy-related proteins like beclin-1 and LC-3. These findings revealed elevated apoptosis and autophagy, along with reduced neuronal differentiation and increased cell mortality in a dose-dependent manner in primary neuronal-like cells derived from human stem cells [36].

Beyond MTT, techniques such as flow cytometry and electron microscopy are employed to analyze cellular alterations and nanoparticle–cell interactions. For scaffold fabrication involving graphene, cytotoxicity was evaluated using immortalized mouse fibroblast cells (3T3/NIH). Graphene was suspended in Dulbecco’s Modified Eagle Medium (DMEM) with 10% fetal bovine serum at a concentration of 0.07 g/mL. The cells were analyzed 24 and 48 h after exposure. The results demonstrated no cytotoxic effect, and a significant increase in cell proliferation was observed after 48 h compared with the control group [61].

Despite GO’s potential applications, comprehensive safety assessments are essential prior to clinical or environmental deployment. Optimizing the physicochemical properties of nanomaterials is crucial to minimizing toxicity while maximizing their functional benefits. At the 26th Unicamp Scientific Initiation Congress, a study was presented evaluating the effects of a graphene oxide scaffold conjugated with polyethylene glycol (6ARM PEG amine (dipentaerythritol) HCl) and polycaprolactone (GO/PEG/PCL), combined with adipose-derived mesenchymal stromal cells (AMSCs), for the repair of localized bladder injuries in rats. Involving 20 Fischer 344 rats, the results demonstrated improved bladder architecture recovery between 8 and 16 weeks post treatment [57,58].

## 4. Results and Discussion

Table 6 summarizes the impact of graphene oxide-based polymeric composites on mesenchymal stem cells, highlighting the effects on cell behavior and the related mechanisms involved. The following sections provide a detailed analysis of the studies referenced in Table 6, elucidating the experimental conditions, observed effects, and underlying mechanisms of action.

In a study conducted by Cerverò-Varona et al. (2023) [87], the objective was to evaluate the effects of glass surfaces coated with graphene oxide (GO) on the phenotype of amniotic epithelial cells (AECs). Gene expression analysis of AECs cultured with GO revealed the upregulation of Snail, Twist, and ZEB transcription factors, as well as the late mesenchymal marker vimentin (VIM), alongside the downregulation of the epithelial marker CYTO8. An increase in the generation of reactive oxygen species (ROS) was also observed in cells cultured on GO-coated coverslips. These findings suggest that GO induced and accelerated the epithelial–mesenchymal transition (EMT), mediated by the intracellular activation of the TGFβ1–SMAD2/3 signaling pathway. Furthermore, enhanced cell migration, characterized by clonal movement, significantly altered cytokine secretion patterns. GO exposure led to increased production of pro-inflammatory cytokines by AECs, thereby promoting a more efficient activation of macrophages within a regenerative microenvironment.

Conversely, a study by Xu et al. (2022) [47] demonstrated that only low concentrations of GO (0.1 and 1 μg/mL) could enhance the proliferation and osteogenic differentiation of mesenchymal stem cells (MSCs). Treatment with these concentrations led to increased levels of active β-catenin and phosphorylation of GSK-3β (*p*-GSK-3β), indicating activation of the Wnt/β-catenin signaling pathway. This pathway is known to favor osteogenic differentiation while inhibiting MSC differentiation into chondrocytes and adipocytes, thereby contributing to bone homeostasis [84]. Alizarin Red staining confirmed an increase in calcium-rich deposits associated with osteoblast differentiation, with increases of 142% and 120% observed at 0.1 and 1 μg/mL GO concentrations, respectively.

Similarly, Halim et al. (2019) [89] demonstrated that low doses of GO nanosheets (0.1 μg/mL) did not significantly affect cell viability but promoted MSC proliferation. Moreover, GO maintained oxidative balance by upregulating antioxidant genes such as manganese superoxide dismutase (MnSOD) and catalase during osteogenic differentiation. These effects were regulated through the activation and nuclear translocation of Forkhead box O1 (FoxO1), which was dependent on the activity of c-Jun N-terminal kinase (JNK). Inhibition of JNK signaling by SP600125 suppressed FoxO1 translocation to the nucleus, subsequently impairing both osteogenic differentiation and the antioxidant defense mechanisms in MSCs.

In a study by Hung et al. (2021) [91], GO-based nanocomposites decorated with gold nanoparticles (GO-Au) were investigated for their biocompatibility and anti-inflammatory effects both in vivo and in vitro. Among the tested materials, GO-Au (x2) demonstrated the highest antioxidant capacity, enhanced cell proliferation, and improved MSC spreading compared with the controls. Additionally, the nanocomposite suppressed immune responses related to the monocyte-to-macrophage transition and inhibited platelet activity, indicating anti-inflammatory potential. Regarding biocompatibility, GO-Au exhibited higher cell viability and reduced cytotoxicity compared with the control group, underscoring its promise as a nanomaterial for biomedical applications.

In a study where mesenchymal stem cells (MSCs) were cultured on alternately patterned matrices of reduced graphene oxide (GO) ultrathin films [89], transforming growth factor β1 (TGF-β1) functioned as an agonist, inducing MSC differentiation into smooth muscle cells (SMCs). This differentiation capacity was consistently maintained and was evidenced by the expression of markers such as calponin and SM22α. Although the aligned MSCs exhibited lower expression of SM22α compared with TGF-β1-induced SMCs, the calponin expression levels were nearly equivalent. Additionally, the expression of the transcriptional coactivator with a PDZ-binding motif (TAZ) on the 100 μm patterned spacing was higher than that on the unpatterned substrate, correlating with an increase in α smooth muscle actin (α-SMA) expression. This strategic approach to directing stem cell fate via specific signaling crosstalk presents a promising platform for cell culture and targeted differentiation.

One study by Karimzadeh et al. (2023) [92] investigated the use of GO to enhance the therapeutic efficacy of MSCs in the treatment of acute kidney injury (AKI). Both studies demonstrated that graphene-based nanostructures reduced serum creatinine (Cr) and blood urea nitrogen (BUN) levels in AKI models. Histopathological analysis revealed increased cell proliferation, reduced apoptosis and necrosis, and a decrease in cyst formation and intratubular debris compared with the control groups. A greater number of renal cells were stained positively for Ki-67 in the group treated with GO in combination with MSCs, strongly suggesting enhanced proliferative activity during tissue repair, as Ki-67 is a marker of cellular proliferation.

These results indicate that GO improves cell–cell and cell–extracellular matrix (ECM) interactions, thereby enhancing the efficacy of MSC transplantation in kidney regeneration.

Jagiełło et al. (2019) [28], in their study on the impact of graphene-based substrates on the biological properties of MSCs derived from human umbilical cord Wharton’s jelly (hUC-MSCs), found that the size of the GO sheets was a critical factor influencing favorable cell culture outcomes. While the hUC-MSCs cultured on a thin GO layer exhibited viability and proliferation rates comparable to those under standard culture conditions, in contrast, the cultured highly reduced GO substrates showed decreased proliferation and increased apoptosis. Moreover, the cells maintained their mesenchymal phenotype, as indicated by the low expression of CD45 and the high expression of CD90 and CD105, consistent with the MSC identity.

**Figure 4:**
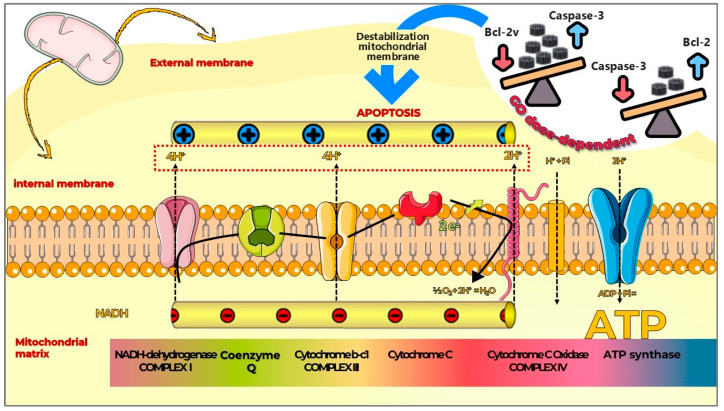
Graphical representation of the apoptosis process induced by high concentrations of graphene oxide (GO) in mesenchymal stem cells (MSCs). The diagram illustrates the destabilization of the mitochondrial membrane, resulting in the release of cytochrome c and activation of the apoptotic cascade. Molecular changes include the downregulation of Bcl-2 and caspase-3, along with the up-regulation of cleaved caspase-3, LC3-II/I, and beclin-1. These changes lead to elevated production of reactive oxygen species (ROS) and a significant loss of mitochondrial membrane potential (MMP). Consistent with the findings of Jagiełło et al. (2019) [28], the biological effects of GO on MSCs are dose-dependent.

In a study by Zhang et al. (2020) [93], high concentrations of GO inhibited cell viability and compromised membrane integrity. This effect was associated with the downregulation of Bcl-2 and caspase-3 and the upregulation of cleaved caspase-3, LC3-II/I, and beclin-1. These molecular changes led to elevated reactive oxygen species (ROS) production and a significant loss of mitochondrial membrane potential (MMP). Consistent with the findings of Jagiełło et al. (2019) [28], the biological effects of GO on MSCs were found to be dose-dependent (Figure 4).

Table 7 comparatively synthesizes the main aspects of the studies included in this review, highlighting the GO and scaffold formulations utilized, the in vitro and in vivo models employed, the key findings regarding MSC modulation, and the inherent limitations of each investigation. This visual compilation facilitates the identification of patterns in the methodologies and results, as well as persistent gaps in the field. The detailed analysis presented in this table serves as a foundation for the in-depth understanding of the molecular mechanisms and signaling pathways that will be discussed in the subsequent subsection.

### 4.1. Molecular Mechanisms and Signaling Pathways of Graphene Oxide-Mediated MSC Modulation in 3D Systems

A deeper understanding of the findings presented in this review reveals that the modulation of mesenchymal stem cell (MSC) behavior and differentiation by graphene oxide (GO)-based nanomaterials in 3D systems transcends a mere surface effect, involving a complex orchestration of intricate molecular mechanisms and intracellular signaling pathways. While many investigations primarily focus on osteogenesis, an integrated analysis demonstrates GO’s capacity to influence multiple cell lineages and biological processes, as comprehensively synthesized below.

One of the centrally observed mechanisms is the direct modulation of intracellular signaling pathways. Low concentrations of GO activate the Wnt/β-catenin pathway in MSCs, evidenced by increased levels of active β-catenin and GSK3β phosphorylation. This activation is pivotal for osteogenic differentiation, while concurrently inhibiting differentiation into chondrocytes and adipocytes, underscoring precise lineage control mediated by GO [47]. Similarly, the TGFβ1-SMAD2/3 signaling pathway has been implicated in the induction of epithelial–mesenchymal transition (EMT) in amniotic epithelial cells cultured on GO-coated surfaces, suggesting GO’s versatility in modulating cellular transformation pathways [88]. In another context, Halim et al. (2019) demonstrated that GO promotes osteogenesis and maintains the oxidative balance of MSCs via activation and nuclear translocation of Forkhead box O1 (FoxO1), a process dependent on c-Jun N-terminal kinase (JNK) activity, a crucial MAPK pathway [90].

Beyond direct intracellular pathway activation, GO exerts significant influence through the modulation of growth factors and extracellular matrix (ECM) protein bioactivity. Its capacity to act as a pre-concentration platform for differentiation-inducing factors is crucial, thereby potentiating their biological effects. A notable example is its role in adipogenic differentiation. GO, by adsorbing insulin (a pivotal mediator in fatty acid synthesis), uniquely allows it to maintain its three-dimensional conformation and bioactivity. This preservation facilitates the effective activation of insulin’s signaling pathway (insulin receptor and subsequent activation of the PI3K/Akt and MAPK/ERK pathways), promoting lipid synthesis. In contrast, graphene (G) induced insulin denaturation through strong π−π interactions, thereby suppressing adipogenesis. This detailed mechanism underscores that the preservation of adsorbed biomolecules’ structural integrity is a critical molecular determinant for cell lineage specification [88].

The physicochemical interaction between GO and MSCs also plays a fundamental role. Enhanced hydrophilicity and increased adsorption of ECM proteins in GO-incorporated scaffolds have been demonstrated, which in turn optimize MSC adhesion and proliferation. These protein interactions, mediated by GO on the cell surface, activate integrins, pivotal receptors for transducing signals from the external environment to the cell interior. Integrin activation, in turn, triggers downstream signaling cascades involving focal adhesion kinase (FAK) and subsequent activation of MAPK pathways (ERK, JNK, and p38), as well as the RhoA/ROCK pathway, which collectively regulate cytoskeletal reorganization, cell adhesion, and gene expression linked to differentiation. Complementarily, the surface topography of GO scaffolds influences cell adhesion and differentiation, suggesting a significant role for mechano-transduction. Pathways such as YAP/TAZ (components of the Hippo pathway) convert mechanical stimuli from the substrate into biochemical signals that regulate MSC fate, influencing their proliferation and differentiation in response to biomaterial stiffness and architecture [94].

Despite these advances in elucidating molecular mechanisms in GO-containing environments, it is crucial to recognize the inherent limitations of planar 2D cell cultures with graphene-based systems. Such cultures, while useful for experimental control, often fail to adequately replicate the complexity of the in vivo cellular microenvironment, which is intrinsically three-dimensional and dynamic. In 2D systems, cells experience simplified signaling gradients and cell–matrix interactions that may not faithfully translate the complex cellular responses observed in native tissues. The lack of 3D cell–cell and cell–matrix interactions, as well as the absence of relevant mechanical cues and mass transport, can result in MSC proliferation and differentiation patterns that differ substantially from those occurring in a physiological context [94].

It is within this context of overcoming the limitations of 2D culture that the exploration of 3D systems based on graphene gains particular relevance for tissue engineering and regenerative medicine. These advanced constructions aim to more faithfully mimic the architectural and biochemical complexity of the tissue environment.

In this regard, the three-dimensional structure and porosity of graphene materials significantly influence cellular response, optimizing attachment, proliferation, and differentiation. Continuous and porous 3D graphene scaffolds (3D-GFs) offer bioactive microenvironments that simulate in vivo conditions, with topographical, chemical, and electrical cues conducive to stem cell differentiation [95].

The exploration of soluble 3D graphene-based scaffolds has proven particularly promising for stem cell culture and differentiation. In studies with bone marrow-derived hMSCs, the use of graphene nanomaterial flakes (G, GO, and pGO) in solutions enabled the efficient formation of cell pellets and micromasses, essential environments for effective chondrogenic differentiation. Notably, while graphene (G) and porous GO (pGO) promoted chondrogenesis, GO demonstrated reduced differentiation with increasing concentrations. This differential effect was attributed to the growth factor pre-concentration capacity (for G and pGO) and the increased diffusional barrier imposed by high GO concentrations, limiting the availability of essential chemical factors [96].

In a hybrid 2D/3D approach, poly(ethylene glycol)-poly(L-alanine) (PEG-L-PA) diblock copolymer hydrogels encapsulating GO or rGO were developed for the culture of tonsil-derived MSCs (TMSCs) [97]. Although the rGO system favored cell proliferation, the hybrid GO/PEG-L-PA system induced a significant increase in the expression of chondrogenic biomarkers, reaffirming GO’s potential for enhanced lineage-specific differentiation in 3D hybrid platforms. Additionally, the incorporation of GO into poly(ethylene glycol) diacrylate (PEGDA) hydrogels created 3D hybrid structures that improved focal adhesion, cell attachment, viability, and survival of hADSCs, culminating in significantly enhanced osteogenic differentiation compared with the controls [97].

**Figure 5:**
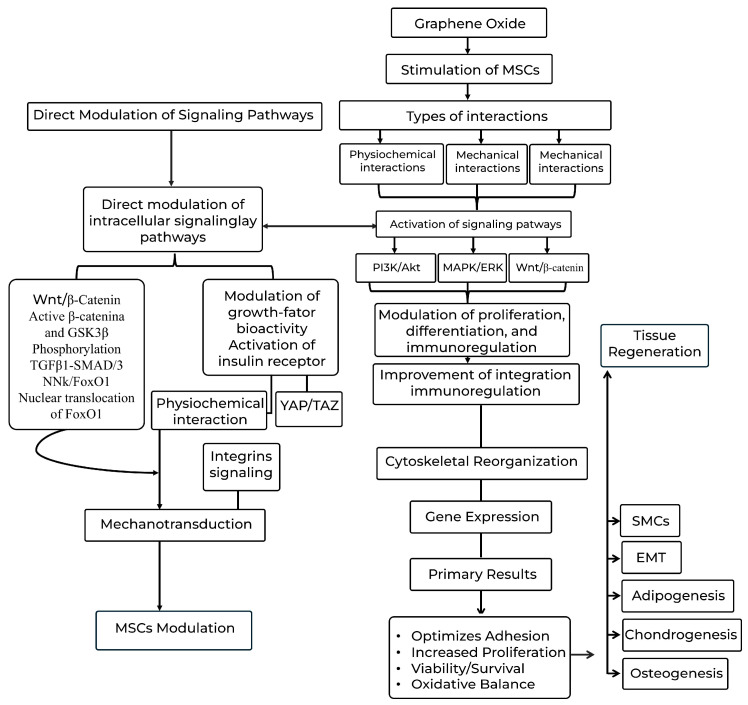
Molecular mechanisms and signaling pathways of graphene oxide-mediated MSC modulation in 3D Systems. The detailed flowchart illustrates the multifaceted interactions between graphene oxide (GO) within 3D biomaterial systems and mesenchymal stem cells (MSCs). It depicts how physicochemical, mechanical, and bioactive interactions with GO stimulate various intracellular signaling pathways (including Wnt/β-catenin, TGF-β1-SMAD2/3, JNK/FoxO1, PI3K/Akt, MAPK/ERK, integrin/FAK/RhoA/ROCK, and YAP/TAZ). These activated pathways collectively lead to cytoskeletal reorganization, altered gene expression, and modulate primary cellular outcomes such as optimized adhesion, increased proliferation, viability, and oxidative balance, ultimately directing MSC lineage-specific differentiation (e.g., osteogenesis, chondrogenesis, adipogenesis, EMT, and SMCs) toward tissue regeneration.

Therefore, the interaction between GO and MSCs in 3D systems is characterized by a complex web of mechanisms encompassing the direct activation of signaling pathways (Wnt/β-catenin and JNK/FoxO1), the modulation of growth factor bioactivity (insulin and its effects on the PI3K/Akt and MAPK/ERK pathways), and the transduction of physicochemical and mechanical signals via the integrins/FAK and YAP/TAZ pathways (Figure 5). Continued exploration of these intricate mechanisms is fundamental for the rational design of more effective GO-based biomaterials capable of precisely and efficiently directing MSC fate in tissue engineering and regenerative medicine applications.

### 4.2. Impact of 3D Printing Parameters and Scaffold Design on Graphene Oxide–MSC Interactions

The advent of three-dimensional (3D) printing, also known as additive manufacturing, has revolutionized the field of tissue engineering by enabling the precise fabrication of highly complex and customized scaffolds that closely mimic the intricate architecture of native tissues [9]. When combined with the unique physicochemical and biological properties of graphene oxide (GO), 3D printing offers an unparalleled platform for designing advanced biomaterials capable of orchestrating mesenchymal stem cell (MSC) behavior with high fidelity [15]. Beyond merely incorporating GO, the specific printing method, the choice of material combinations, and the deliberate design of architectural parameters exert a profound influence on how GO interacts with MSCs, ultimately dictating cellular fate and tissue regeneration outcomes. This section meticulously examines how these diverse factors collectively contribute to shaping the biological responses of MSCs, thereby impacting the efficacy of GO-based scaffolds in regenerative medicine applications.

#### 4.2.1. Specific 3D Printing Methods and Their Implications for GO–MSC Interactions

Various additive manufacturing techniques are employed to create GO-integrated scaffolds, each presenting distinct advantages and challenges that impact MSC viability and GO functionality.

Extrusion-Based Bioprinting: This method involves extruding a bioink (a mixture of cells, biomaterials, and sometimes GO) through a nozzle to create desired structures layer by layer [68]. Its versatility in handling a wide range of viscous materials and scalability makes it highly attractive. However, MSCs encapsulated within GO-loaded bioinks may experience significant shear stress during extrusion, potentially affecting their viability and post-printing functionality, which can compromise initial cell seeding efficiency and long-term tissue maturation [57]. Conversely, the fibrous nature of extruded constructs can induce cell alignment and direct differentiation, influenced by the anisotropic cues provided by the GO within the matrix, thereby guiding cellular organization crucial for mimicking native tissue anisotropy [98].

Digital Light Processing (DLP) and Stereolithography (SLA): These light-based techniques involve the photopolymerization of a liquid resin (containing GO or encapsulated cells) layer by layer using UV or visible light [84]. DLP and SLA offer superior resolution and enable the creation of highly intricate micro-architectures and complex internal geometries [66]. The primary challenges include potential cytotoxicity from photoinitiators and limitations in bioink viscosity. When GO is incorporated, it can influence light penetration and curing efficiency [84]. The precise control over pore size and micro-patterns achieved through these methods directly impacts MSC adhesion, spreading, and subsequent differentiation by providing specific topographical cues and mediating mechanotransduction, leading to enhanced osteogenesis or chondrogenesis, depending on the designed pattern [96].

Inkjet Bioprinting: This non-contact method deposits pico- or nanoliter droplets of bioink onto a substrate [47]. It typically results in high cell viability due to minimal mechanical stress. However, it is limited to low-viscosity bioinks and may suffer from nozzle clogging when incorporating particles like GO at higher concentrations [47]. For GO/MSC systems, inkjet bioprinting can ensure a more uniform distribution of GO throughout the scaffold at lower concentrations, promoting homogeneous cellular responses and potentially influencing nutrient delivery within the scaffold, which is vital for sustained cell survival and metabolic activity in 3D constructs [68].

#### 4.2.2. Material Combinations with Graphene Oxide in 3D Printing

The choice of matrix material profoundly affects the mechanical properties, degradation profile, and overall biocompatibility of GO-integrated 3D printed scaffolds, thereby influencing MSC behavior.

GO with Hydrogels: Hydrogels (e.g., gelatin methacrylate (GelMA), poly(ethylene glycol) diacrylate (PEGDA), alginate, and hyaluronic acid) are highly favored for bioprinting due to their high water content and resemblance to a native ECM [97]. GO incorporation significantly enhances the mechanical strength, electrical conductivity, and biological activity of these hydrogels. This combination can create a more biomimetic environment, promoting MSC adhesion, proliferation, and specific lineage differentiation by modulating scaffold stiffness and presenting bioactive cues that actively guide cell fate [66,69] (e.g., studies have shown increased osteogenesis and matrix mineralization in MSCs cultured on GO-GelMA hydrogels compared with hydrogels without GO).

GO with Thermoplastics: Thermoplastic polymers (e.g., polycaprolactone (PCL), polylactic acid (PLA), and poly(lactic-co-glycolic acid) (PLGA)) offer excellent mechanical integrity and tunable degradation rates, making them suitable for load-bearing applications [70]. GO can be blended with these polymers to improve their mechanical properties, enhance hydrophilicity, and confer bioactivity. 3D-printed thermoplastic scaffolds incorporating GO can provide robust platforms that support long-term MSC culture and differentiation, with the GO influencing cell attachment and acting as nucleation sites for tissue formation, thereby accelerating and enhancing new tissue deposition within the scaffold structure [66,70,98].

GO with Bioceramics: Bioceramics (e.g., hydroxyapatite (HA) and β-tricalcium phosphate (β-TCP)) are osteoconductive and osteoinductive, making them ideal for bone regeneration [96]. Incorporating GO into ceramic bioinks or composites can improve printability, enhance mechanical strength, and promote MSC osteogenic differentiation by providing a synergistic effect, leading to superior bone repair outcomes. The GO can facilitate better cell–material interactions and potentially influence the local ionic environment to drive differentiation, creating a more favorable osteoinductive niche for MSCs.

#### 4.2.3. Architectural Design Parameters and Their Influence on GO–MSC Interactions

Beyond the material composition, the precise architectural control offered by 3D printing is critical for directing MSC fate.

Pore Size and Interconnectivity: The optimal pore size facilitates nutrient and oxygen transport, waste removal, and cell infiltration within the 3D scaffold [9]. Highly interconnected porous networks, achievable through 3D printing, are crucial for uniform cell distribution and vascularization, which directly impacts long-term cell viability and tissue integration in vivo [66]. When GO is integrated, it can further influence pore morphology and surface properties, impacting MSC migration and access to bioactive sites within the scaffold, thereby driving differentiation pathways such as osteogenesis or chondrogenesis For instance, specific pore sizes (e.g., typically ranging from 100 to 500 µm for bone) are known to optimize MSC proliferation and subsequent differentiation, a factor that GO can further enhance through its bioactivity within these pores [94].

Surface Topography and Micro-Patterns: Three-dimensional printing allows for the creation of intricate surface features, from micro-ridges to specific patterns, which can profoundly influence MSC behavior. GO’s inherent roughness and its ability to be precisely patterned within a 3D matrix can dictate MSC adhesion, spreading, and even cellular alignment [99]. These topographical cues activate specific mechanotransduction pathways, such as the YAP/TAZ pathway, converting physical stimuli into biochemical signals that regulate gene expression and lineage commitment toward osteogenic or chondrogenic phenotypes, ultimately directing the desired tissue formation [93].

Mechanical Stiffness (Rigidity): The mechanical properties of a scaffold, particularly its stiffness, are known to be critical determinants of MSC fate. Three-dimensional printing allows for precise tuning of the scaffold stiffness by controlling the material composition (e.g., GO concentration), crosslinking density, and architectural design (e.g., strut thickness and pore size) [100]. GO significantly enhances the stiffness of many polymeric matrices. This tunable rigidity directly influences MSC mechanosensing, promoting adipogenesis on softer substrates, myogenesis at an intermediate stiffness, and osteogenesis on stiffer constructs, by modulating focal adhesion signaling and cytoskeletal tension, thereby dictating the cell’s differentiation pathway [8].

Resolution and Anisotropy: High-resolution 3D printing enables the fabrication of scaffolds with features on the micro scale, closely mimicking the complexity of in vivo cellular niches. The ability to create anisotropic structures (e.g., aligned channels or fibers) can guide MSC migration and differentiation, which is particularly relevant for regenerating anisotropic tissues like muscle, nerve, or bone [57]. GO, when aligned within such structures, can further enhance the anisotropic cues, promoting specific cell alignment and differentiation patterns, which is critical for mimicking the organized architecture of native tissues and ensuring functional regeneration [92].

In summary, the precise control over 3D printing parameters, including the choice of printing method, material combinations, and architectural design, allows for an unprecedented level of control over the cellular microenvironment within GO-based scaffolds. This intricate interplay between GO’s intrinsic properties and the engineered scaffold features ultimately dictates MSC behavior, guiding their adhesion, proliferation, migration, and differentiation toward desired lineages, thereby directly impacting the success of tissue engineering and regenerative medicine strategies.

## 5. Gaps in the Literature and Future Directions

Gaps in the literature and future directions for research on graphene oxide (GO)-based nanomaterials and their cytotoxic properties are crucial areas for deepening our understanding and improving the safety and efficacy of these materials in various applications. The following highlights the main gaps and future research directions.

### 5.1. Gaps in the Literature

Although the existing literature has contributed to understanding the general effects of graphene oxide (GO) and its derivatives, the exact mechanisms underlying their cytotoxicity remain insufficiently elucidated. GO toxicity is frequently associated with phenomena such as reactive oxygen species (ROS) generation, membrane disruption, and metabolic interference. However, these hypotheses still lack comprehensive experimental validation. More in-depth investigations employing high-throughput techniques—such as proteomics, metabolomics, and high-resolution microscopy—are essential to map the molecular and cellular pathways affected by GO [46,55,57].

Most current studies address the acute toxicity of GO, leaving critical questions about its long-term effects on mesenchymal stem cell (MSC) viability and functionality unresolved. Accumulation of GO in tissues and organs may lead to chronic cellular alterations, including potential carcinogenicity or immunotoxicity. There is an urgent need for experimental models that simulate repeated or prolonged exposure, supported by histopathological analysis, biodistribution studies, and systemic toxicological assessments to clarify the long-term implications for cell survival and therapeutic efficacy [81].

The predominance of studies employing specific cell lines or isolated tissues limits the extrapolation of findings. GO may exhibit distinct interactions with epithelial, neuronal, endothelial, and immune cells, as well as complex tissues such as the brain and liver. Expanding research to include primary human cells, organotypic models, and co-culture systems may uncover critical variations in GO toxicity across diverse biological contexts [6,36,71,101].

The relationship between GO and the immune system remains underexplored. Existing studies indicate that GO can elicit inflammatory responses through macrophage activation and cytokine release. However, broader and long-term consequences for both innate and adaptive immunity, particularly regarding chronic inflammation or immunomodulation by GO-MSC constructs in vivo, remain unclear. Focused investigations are required to identify specific immune receptors involved and elucidate the molecular mechanisms underlying immune modulation by GO, addressing how these responses might impact the persistence and therapeutic outcome of MSCs in regenerative applications [61].

Considerable variability in the methodologies used to assess GO cytotoxicity has led to inconsistent and sometimes contradictory findings in the literature. This lack of standardization hinders comparative analysis and complicates the establishment of regulatory guidelines. Developing standardized protocols—including reference concentrations, relevant cell models, and consistent analytical parameters—will be pivotal for advancing the field [72].

In both environmental and biomedical settings, GO is often encountered in combination with other agents such as heavy metals, organic pollutants, or pharmaceutical compounds. Nevertheless, studies examining the synergistic or antagonistic effects of these combinations remain limited. Such investigations are crucial to anticipate potential risks and guide the safe and effective use of GO in complex environments [6,36,71].

While in vitro studies offer valuable mechanistic insights, they cannot replicate the complexity of living organisms. In vivo animal models that simulate realistic human exposure scenarios are vital to assess systemic effects and potential complications associated with GO. However, a significant gap remains in available in vivo studies regarding the detailed and long-term assessment of functional tissue integration following the implantation of GO-based scaffolds loaded with MSCs. While evidence for tissue regeneration exists, comprehensive data on the mechanical, structural, and physiological integration of the newly formed tissue with the host and how this functional integration translates into sustained therapeutic outcomes in various physiological contexts are often limited. Research involving mammalian models that evaluate parameters such as organ-specific toxicity, systemic inflammation, immune modulation, and robust functional integration assays will provide critical information [71].

Understanding the biocompatibility and biodistribution of GO is fundamental to evaluating its potential for biomedical applications. Emerging evidence suggests that GO can accumulate in the liver, spleen, and kidneys. However, the consequences of such biodistribution over the long term remain largely unknown. Further research is required to investigate the body’s ability to metabolize or excrete GO and its derivatives, as well as their possible bioaccumulation and toxicity [81].

### 5.2. Future Directions

Structural modifications and chemical functionalization strategies for graphene oxide (GO) may significantly reduce its cytotoxicity. For instance, the incorporation of antioxidant functional groups or biocompatible coatings can diminish reactive oxygen species (ROS) generation and enhance the compatibility of GO with cellular and tissue environments [54].

The application of three-dimensional (3D) bioprinted tissue models, organoids, and organ-on-a-chip systems offers more physiologically relevant platforms to evaluate the biological effects of GO. These advanced models simulate complex multicellular environments and enable comprehensive analyses of cellular responses [24].

Detailed studies of the cellular and molecular mechanisms underlying GO toxicity are essential. These may include the investigation of intracellular signaling pathways, disruptions in redox homeostasis, and potential epigenetic modifications. A deeper understanding of these mechanisms is critical for the development of mitigation strategies and safer nanomaterials [8,24,100].

The increasing application of GO raises concerns about its environmental release and potential impacts on ecosystems. Ecotoxicological studies should assess the environmental persistence of GO, its bioaccumulation, and its effects on aquatic and terrestrial organisms. These data will be crucial to inform regulatory policies on GO use and disposal [102].

The establishment of specific regulatory frameworks for GO usage is urgently needed to mitigate associated risks. This includes the development of standardized safety assessment protocols, handling and storage procedures, and labeling requirements for materials containing GO [53].

The interaction between GO and the human and environmental microbiome represents an emerging research area. Preliminary evidence suggests that GO may alter microbial communities, with possible implications for host health and ecological balance. Further investigation is required to characterize these interactions and understand their long-term effects [57,58,103].

GO holds considerable promise for clinical applications such as targeted drug delivery and gene therapy. However, rigorous safety evaluations, including preclinical and clinical trials, are essential to ensure a favorable balance between therapeutic efficacy and potential toxicity [72].

Interdisciplinary collaboration among researchers in nanotechnology, cell biology, medicine, and engineering will be fundamental to the responsible development of GO-based technologies. Integrated approaches will foster innovation and contribute to safer, more effective biomedical and environmental applications [100].

#### Emerging Alternatives and Comparative Perspectives in Scaffold Design

While graphene oxide (GO) has undeniably demonstrated remarkable potential in orchestrating MSC behavior within 3D scaffolds, a comprehensive perspective on the field necessitates critically examining emerging alternative materials and strategies. Future research must also explicitly address GO’s inherent limitations and comparative disadvantages (beyond those pertaining to safety and long-term biological interactions already discussed in Section 5.1).

The landscape of advanced biomaterials for tissue engineering is rapidly expanding, with several emerging alternatives offering distinct characteristics that may complement or, in specific applications, even surpass those of GO. For instance, other two-dimensional (2D) materials, such as transition metal dichalcogenides (e.g., MoS2 and WS2) and MXenes (e.g., Ti3C2Tx), are gaining significant attention. These materials possess unique electronic, mechanical, and surface chemistries that could offer novel platforms for MSC modulation, potentially providing tailored cues for specific differentiation pathways or enhanced biodegradability profiles compared with GO [12]. Beyond 2D nanomaterials, the continued development of advanced hydrogels and polymeric scaffolds, precisely engineered with specific pore architectures, tunable mechanical properties, and controlled release of bioactive factors (even without graphene derivatives), represents a parallel and competitive avenue. These alternatives often present more established regulatory pathways and simplified synthesis methods, which can expedite clinical translation [12].

Furthermore, it is crucial to acknowledge certain intrinsic drawbacks and persistent challenges associated with GO-based scaffolds that, despite ongoing research efforts, remain significant hurdles. One notable concern is the inherent variability in GO synthesis protocols, which can lead to inconsistencies in material properties across different batches and suppliers. This lack of standardization complicates direct comparability between studies and poses a substantial challenge for reproducible manufacturing on a clinical scale. Another drawback pertains to precise control over the long-term degradation kinetics of GO within complex 3D polymeric or ceramic matrices. While some degradation pathways have been identified, achieving a predictable and tunable degradation rate that precisely matches the pace of new tissue formation, without generating harmful byproducts over extended periods, remains a complex challenge [66]. These considerations highlight the need for continued innovation not only in optimizing GO-based systems but also objectively evaluating their performance against and potential synergistic integration with the diverse array of rapidly advancing alternative biomaterials and fabrication techniques.

### 5.3. Safety and Biocompatibility of Graphene Oxide in 3D Systems: Current Insights and Risk Mitigation

While graphene oxide (GO) and its derivatives exhibit immense promise across various biomedical applications, particularly in tissue engineering and regenerative medicine through 3D systems, a comprehensive understanding of their safety and biocompatibility is paramount for successful clinical translation. Despite the acknowledged gaps in the literature, recent advancements have provided more concrete insights into the in vivo toxicity profile and established emerging best practices for minimizing associated risks. The complex interplay between GO’s physicochemical properties, the administration route, dosage, and specific biological environment dictates its ultimate biocompatibility.

#### Key Factors Influencing Graphene Oxide Biocompatibility and Toxicity In Vivo

The biological interactions and potential toxicity of GO in vivo are highly dependent on its intrinsic physicochemical characteristics and how it is presented to biological systems.

Size and Lateral Dimensions: Smaller GO sheets tend to exhibit different cellular internalization rates, biodistribution patterns, and elimination kinetics compared with larger sheets. Smaller flakes may accumulate more readily in certain organs or cross biological barriers more easily, while larger flakes might experience aggregation or more rapid clearance [96].

Oxidation State and Surface Functional Groups: The degree of oxidation, presence of carboxyl, hydroxyl, and epoxy groups, and their spatial distribution significantly influence GO’s hydrophilicity, charge, and reactivity. These functional groups determine protein adsorption profiles, cellular uptake mechanisms, and subsequent intracellular processing or degradation, directly impacting immunogenicity and toxicity [16].

Purity: Residual impurities from synthesis processes (e.g., metal catalysts and strong oxidants) can contribute to the observed toxicity, making highly purified GO essential for biomedical applications [93].

Number of Layers: Monolayer GO sheets typically display distinct biological responses compared with multi-layered GO or graphene stacks, influencing cellular interactions and degradation [71].

Surface Charge (Zeta Potential): The surface charge dictates colloidal stability, aggregation tendencies, and interactions with cell membranes and circulating proteins, profoundly affecting biodistribution and cellular fate in vivo [63].

Concentration and Dosage: As with any material, the dose of GO administered is a critical determinant of its toxicity. High concentrations often lead to acute toxic responses, while lower, therapeutic doses may be well tolerated or even beneficial [47].

Route of Administration: The entry point into the biological system (e.g., intravenous, intraperitoneal, oral, subcutaneous, or direct implantation within a 3D scaffold) dictates the initial biodistribution, immune exposure, and potential organ accumulation (e.g., intravenous administration often leads to accumulation in reticuloendothelial system organs) [45].

Degradation and Stability In Vivo: The stability of GO and its degradation products within the biological milieu are crucial. Enzymatic degradation by myeloperoxidase (MPO) and other enzymes has been demonstrated, impacting GO’s long-term persistence and clearance [66].

### 5.4. Translational Challenges and Regulatory Landscape for Graphene Oxide-Based Scaffolds

Despite the remarkable scientific advancements in graphene oxide (GO)-based scaffolds for mesenchymal stem cell (MSC) modulation, their successful translation from the laboratory bench to clinical application faces substantial hurdles that extend beyond fundamental science. Navigating the complex regulatory frameworks, overcoming commercialization challenges, and progressing through rigorous preclinical and clinical trials are critical steps for these innovative biomaterials to impact patient care.

#### 5.4.1. Emerging Regulatory Frameworks for Nanomaterials in Healthcare

The regulatory landscape for nanomaterials and combination products (e.g., scaffolds with cells, drugs, or growth factors) is evolving, presenting unique challenges for GO-based scaffolds.

Classification of Nanomaterials: Regulatory bodies like the U.S. Food and Drug Administration (FDA) and the European Medicines Agency (EMA) grapple with defining and classifying nanomaterials due to their unique size-dependent properties. GO-based scaffolds may be classified as medical devices, drugs, biologics, or combination products, each with distinct regulatory pathways and requirements [73]. This ambiguity can prolong the approval process.

FDA Approach (USA): The FDA adopts a “case-by-case” approach for nanomaterials, assessing risks and benefits based on the specific product and intended use [31]. For GO-based scaffolds, this involves rigorous scrutiny of manufacturing processes, detailed physicochemical characterization, in vitro and in vivo biocompatibility and safety data, and preclinical efficacy data. If the scaffold incorporates cells, it may fall under “human cell and tissue products” (HCT/Ps) or “cellular and gene therapy products,” adding further regulatory layers.

EMA Approach (Europe): The EMA utilizes the Medical Devices Regulation (MDR) and the Advanced Therapy Medicinal Products (ATMPs) regulation for products with nanomaterials. ATMPs (which include cell-based therapies like MSCs on scaffolds) have specific guidelines, and GO as a component would be subject to stringent safety and quality requirements [26]. The Registration, Evaluation, Authorisation and Restriction of Chemicals (REACH) regulation also influences the use of nanomaterials, though primarily for industrial applications.

Specific Regulatory Challenges for GO: The lack of a universally harmonized definition for “nanomaterial” across global agencies, the complex and variable nature of GO synthesis (leading to batch-to-batch variability), and ongoing debates regarding long-term in vivo fate and potential degradation products [73] all contribute to regulatory uncertainty. There is a pressing need for standardized test methods and clear guidelines specifically for graphene-based materials in biomedical applications.

#### 5.4.2. Commercial Challenges and Market Adoption

Translating GO-based scaffolds into commercially viable products involves significant non-scientific hurdles.

Scalability of Production: Producing high-quality, consistent GO and fabricating complex 3D scaffolds at a scale sufficient for commercialization remains a major challenge. Current synthesis methods for GO can be energy-intensive and yield variable products, hindering large-scale, reproducible manufacturing [98]. Scaling up 3D printing of intricate, cell-laden constructs while maintaining sterility and quality is also complex and costly.

Cost-Effectiveness: The research and development costs, regulatory approval expenses, and manufacturing costs of advanced GO-based scaffolds can be substantial. For market adoption, the final product must demonstrate clear superiority and cost-effectiveness compared with existing treatments or alternative biomaterials [59].

Intellectual Property (IP): The rapidly evolving and crowded patent landscape for graphene technologies and their biomedical applications can create significant intellectual property challenges, potentially leading to costly litigation or limiting market entry for new innovators [70].

Market Acceptance and Competition: Despite their potential, novel biomaterials, particularly those involving nanotechnology, may face skepticism from clinicians and patients due to safety concerns or unfamiliarity. Competition from established biomaterials (e.g., collagen, ceramics, and synthetic polymers) and existing surgical procedures requires strong evidence of superior efficacy, safety, and long-term benefits [92].

Reimbursement Policies: Obtaining favorable reimbursement policies from healthcare providers and insurance companies is crucial for market penetration, which often requires robust evidence of clinical utility and health economic benefits.

#### 5.4.3. Preclinical and Clinical Trial Landscape

The pathway from preclinical validation to clinical application for GO-based scaffolds is lengthy, costly, and highly regulated.

Extensive preclinical development (mandatory) before human trials is required to demonstrate the safety and preliminary efficacy of GO-based scaffolds in vivo:

- In Vivo Efficacy Studies: These involve using relevant animal models (e.g., rodents or large animals like pigs or sheep for orthopedic applications) to evaluate the scaffold’s ability to promote tissue regeneration, integrate with host tissue, and restore function [5]. These studies must mimic the human disease state as closely as possible, with a particular emphasis on assessing not just tissue formation but its functional integration and long-term stability within the native biological environment.
- Long-Term Safety Studies: Beyond acute toxicity, studies assessing biodistribution, degradation, and potential long-term systemic or local toxicities over extended periods (e.g., from 6 months to 1 year or more) are critical, including the sustained viability and function of MSCs within the implanted constructs [102].
- Immunogenicity and Biocompatibility: Comprehensive evaluation of immune responses, inflammatory reactions, and foreign body responses to the implanted scaffold, clarifying both acute and chronic immune responses that might influence the long-term viability of the MSCs and the successful integration of the scaffold are required [81].
- Good Laboratory Practice (GLP): All preclinical studies intended for regulatory submission must adhere to GLP guidelines to ensure data quality, integrity, and reproducibility [50].

In the current clinical trial landscape, as of mid-2025, the clinical trial landscape for GO-based scaffolds, particularly those involving MSCs for specific tissue engineering applications, remains nascent. While individual graphene-related materials might be in early-stage trials for other indications (e.g., diagnostics and drug delivery), rather few if any human clinical trials are publicly registered and actively recruiting for complex GO-based 3D scaffolds combined with MSCs for tissue regeneration. Most research remains in the preclinical phase [49].

Challenges in clinical translation include the following:

- Safety Data: The biggest hurdle is establishing long-term safety, especially given the concerns surrounding nanomaterials and potential persistence or degradation products, which directly impact long-term MSC viability in vivo.
- Efficacy in Humans: Demonstrating robust and reproducible efficacy in complex human physiological environments, which are often different from animal models, particularly regarding the long-term functional integration of engineered tissues and the sustained therapeutic effect of MSCs, is a significant challenge.
- Trial Design: Designing rigorous clinical trials with appropriate endpoints, patient cohorts, and control groups is complex and resource-intensive.
- Ethical Considerations: Especially for cell-based combination products, ethical considerations related to stem cell use are paramount.

## 6. Conclusions

Based on the studies presented, this review concludes that mesenchymal stem cells (MSCs) play a pivotal role in regenerative medicine, with promising potential to treat a wide range of conditions due to their ability to proliferate, differentiate, and exert immunomodulatory effects. MSCs, found in various human tissues, can differentiate into multiple cell lineages, making them valuable tools for tissue regeneration.

The extracellular matrix (ECM) and biomaterials such as graphene oxide (GO) are essential for the effective cultivation and functional differentiation of MSCs, as they provide structural support and modulate cellular behavior through the activation of various signaling pathways. Despite significant progress in applying MSCs across several areas of regenerative medicine, there remain important gaps in the understanding of their interactions with GO.

The chemical interactions between GO and MSCs, as well as the influence of polymer composites on MSC gene expression and cellular function, represent areas that require further investigation. Future research should aim to elucidate the specific molecular mechanisms through which GO modulates MSC behavior and differentiation. Moreover, long-term studies using in vivo models are necessary to assess the biocompatibility and safety of GO-based systems, ensuring their suitability for clinical application.

This review emphasizes the need for continued research to explore the therapeutic potential of GO in combination with MSCs, aiming to contribute to the development of more effective and safe regenerative strategies. A deeper understanding of these mechanisms is crucial for advancing MSC-based therapies.

Ultimately, this study provides a strong foundation for future investigations in this promising field, underlining the importance of interdisciplinary and collaborative approaches to optimize the therapeutic potential of MSCs and GO while minimizing the associated risks.

## Figures and Tables

**Table 1:** Growth factors, cytokines, and biologically active molecules.

Category	Molecule	Function	References
Growth Factors	Platelet-Derived Growth Factor (PDGF)	Stimulates cell proliferation and migration, key in wound healing and tissue regeneration	[33]
	Vascular Endothelial Growth Factor (VEGF)	Promotes angiogenesis, essential for tissue repair and regeneration	[34]
	Transforming Growth Factor Beta (TGF-β)	Regulates cell proliferation, differentiation, and extracellular matrix production	[35]
	Epidermal Growth Factor (EGF)	Stimulates cell proliferation and differentiation, participates in tissue repair	
	Insulin-Like Growth Factor 1 (IGF-1)	Supports cell survival, growth, and differentiation, contributes to bone and cartilage regeneration	[37]
	Fibroblast Growth Factor (FGF)	Involved in tissue regeneration and angiogenesis by promoting cell proliferation and differentiation	[38]
	Bone Morphogenetic Protein 2 (BMP-2)	Promotes cartilage and bone formation, involved in osteogenesis and bone homeostasis	[2,3]
	Bone Morphogenetic Protein 4 (BMP-4)	Involved in bone and cartilage development, muscle development, and fracture repair	[4,5]
	Bone Morphogenetic Protein 7 (BMP-7)	Induces osteoblast differentiation, involved in bone homeostasis and kidney development	[6,7]
Cytokines	Interleukin-6 (IL-6)	Regulates inflammation and modulates the immune response	[43]
	Interleukin-10 (IL-10)	Anti-inflammatory cytokine, central in immune regulation	[44]
	Tumor Necrosis Factor Alpha (TNF-α)	Key regulator of inflammation, with both pro-inflammatory and regenerative properties	[45]
Biologically Active Molecules	Prostaglandins	Lipid-derived signaling molecules involved in inflammation modulation and injury response	[46]
	Matrix Metalloproteinases (MMPs)	Enzymes that degrade extracellular matrix components, facelifted tissue remodeling and wound healing	[55]
	Soluble Cytokines	Including chemokines, which recruit inflammatory cells to injury sites and orchestrate local immune responses	[48]
	Exosomes	Nano-sized extracellular vesicles secreted by MSCs, mediating intercellular communication and influencing immune and inflammatory responses	[49]
Signaling Pathways	Wnt-3	Regulates cell fate decisions, involved in mesenchymal stem cell differentiation	[8,9]
	Wnt-4	Plays a role in organogenesis, sex determination, and mammary gland development	[10,11]
	β-Catenin	Central mediator of the canonical Wnt signaling pathway, regulates gene expression	[19,27]
	Wnt/β-Catenin Pathway	Integrates signals from various pathways, regulates stem cell pluripotency and cell fate decisions	[29,30,56]

**Table 2:** Summary of the main scaffold fabrication methodologies used for GO/PLLA composites, detailing their advantages and disadvantages in the context of tissue engineering.

Fabrication Method	Basic Principle	Advantages of GO/PLLA Composites	Disadvantages or Limitations of GO/PLLA Composites	References
3D Printing (Extrusion and FDM)	Deposits material layer by layer	- High geometric precision and control over internal architecture (porosity and geometry) - Possibility of incorporating cells and biomolecules (bioprinting) - Good reproducibility	- Limited resolution at the nanometer scale (for extremely small pores) - Material viscosity can be challenging - Potential mechanical or thermal stress on cells (bioprinting)	[50]
3D Printing (SLA and DLP)	Light-induced polymerization of photoinitiator in resin	- High resolution and fine details, allowing for complex structures - Compatible with certain photoinitiator-functionalized GOs - Less mechanical stress for cells (if used in bioprinting)	- Restriction to photopolymerizable materials - Requires removal of residual photoinitiators - High equipment and material costs	[63]
Electrospinning	Submicron and nanometer fibers formed by electric field	- Produces membranes with high surface area and interconnected porosity - Mimics natural ECM structure - Allows fiber orientation - Suitable for incorporating nanomaterials like GO	- Difficulty in controlling 3D porosity and thickness (usually forms as 2D or 2.5D) - Low mechanical strength in some configurations - Can be challenging for large-volume production	[64]
Particulate Leaching	Material encapsulated with removable particles	- Simple and low cost - Allows high porosity and interconnected pores - Control over pore size by porogen size	- Difficulty in achieving uniform porosity and complex architecture - Porogen residues can be toxic - Less control over the final scaffold shape	[65]
Freeze-Drying	Freezing followed by solvent sublimation	- Produces highly porous scaffolds with interconnected pores - Suitable for heat-sensitive materials (GO/PLLA) - Simple and low-cost	- Limited control over pore size and geometry (irregularity) - Often low mechanical properties - Difficult controlling pore orientation	[66]
Solvent Casting and Evaporation	Solvent evaporation from a polymer solution	- Simple and low-cost for films or thin layers - Good for GO/PLLA composites	- Difficulty in creating complex 3D structures and interconnected pores - Solvent residues can be toxic to cells	[9]
Self-Assembly	Spontaneous organization of components into structures	- Allows nanoscale structures with high precision and order - Mimics natural biological processes - Can form porous hydrogels	- Difficult to control macro-structure and scaffold scale - High cost of precursors - Complex process for large-scale fabrication	[67]
Spheroid and Microsphere Formation	Cell or polymeric agglomeration into micro-structures	- Creates modular units for tissue engineering - Can be used for delivering cells or growth factors - Potential for in situ vascularization upon implantation	- Limited control over spheroid internal morphology - Challenges in mechanical stability when used as isolated scaffolds - Difficulty in fusing into larger, complex structures	[68]
Gelling and In Situ Hydrogel Formation	Transformation of solution into gel at the site of interest (or in vitro)	- Allows gentle encapsulation of cells (bioprinting) and biomolecules - High similarity to natural ECM in terms of water content - Injectable for minimally invasive applications	- Low mechanical strength for tissues requiring support - Degradation rate is difficult to control precisely - Some gelling initiators may be cytotoxic for GO/PLLA	[69]
Melt Processing	Processing polymers above their melting temperatures	- Wide variety of techniques (injection molding and extrusion) - High production capacity and cost-effectiveness - Improves GO dispersion in some cases	- High temperatures can degrade GO (functionalities) or biomolecules - Difficulty in creating interconnected porosity without secondary porogens - Less suitable for cell incorporation	[70]

**Table 3:** Advantages of GO/PLLA composites for drug release efficiency.

Drug Release Characteristics	Specific Advantages of GO/PLLA Composites	References
High Loading Capacity and Versatility	- GO has a large surface area and functional groups that allow a high loading capacity of hydrophilic and hydrophobic drugs via π-π stacking interactions, hydrogen bonding, and electrostatic adsorption	[71]
Specificity and Targeting	- GO can be functionalized to target the composite to specific locations (cells and tissues), minimizing systemic effects - Systems can be responsive to stimuli (pH, enzymes, and light), allowing for controlled and on-demand release	[44]
Controlled and Sustained Release	- The PLLA matrix offers sustained drug release, useful for long-term treatments and reduced dosing frequency - The release profile can be adjusted by the GO/PLLA ratio and material architecture	[50]
Biocompatibility and Biodegradability	- Both GO (in appropriate concentrations) and PLLA are biocompatible, reducing adverse reactions - PLLA’s biodegradability ensures safe disposal of the material after its function	[72]
Stability	GO/PLLA combination can protect the drug from degradation and optimize bioavailability	[53]

**Table 4:** Disadvantages and challenges of GO/PLLA composites in drug delivery.

Drug Release Characteristics	Disadvantages and Specific Challenges of GO/PLLA Composites	References
Nonspecific Adsorption and Complexity	- Potential for nonspecific adsorption of proteins and other biomolecules on GO, affecting drug release or causing unwanted interactions - Functionalization for high specificity can increase manufacturing complexity and cost	[73]
Loading Limitations	- Loading capacity may be limited by suboptimal drug–polymer–GO interactions or solubility restrictions - GO aggregation can reduce the available surface area	[74]
Challenges in Controlled Release	- PLLA degradation rates can be too slow for some applications, necessitating modifications - Precise control over release kinetics can be challenging in complex biological environments - Initial “burst release” can occur and may be undesirable for certain treatments	[75]
Long-Term Biosafety Concerns	- Although biocompatible, the long-term safety and metabolic fate of GO, PLLA, and their degradation products in vivo still require further, more in-depth studies - PLLA degradation can generate acidic byproducts - GO cytotoxicity can be an issue at high concentrations or with certain cell types	[76]
Homogeneity and Reproducibility	- Difficulty in ensuring homogeneous GO dispersion within the PLLA matrix, which directly impacts the consistency of drug release	[77]

**Table 5:** Factors influencing the cytotoxicity of graphene oxide (GO)-based nanomaterials.

Factor	Description	Effect on Cytotoxicity	References
Particle Size	Smaller particles present a larger surface area for interactions.	Increases membrane penetration and tissue infiltration, enhancing toxic effects.	[82]
Particle Shape	Spherical, lamellar, or elongated morphologies.	Different shapes affect endocytosis and membrane dynamics, potentially causing mechanical damage.	[16]
Degree of Oxidation	Presence of oxygen-containing functional groups such as epoxides and hydroxyls.	It can trigger oxidative stress and metabolic disturbances.	[59]
Functionalization	Chemical modification to reduce intrinsic toxicity.	Improves biocompatibility and stability in aqueous media.	[62]
Dose and Concentration	Higher concentrations are associated with increased toxicity.	Increases oxidative stress, inflammatory responses, and cell death.	[51]
Exposure Time	Duration of exposure to nanomaterials.	Prolonged exposures increase the likelihood of intracellular accumulation and cumulative damage.	[53]
Cell Type	Metabolic and cell cycle differences among cell types.	Influences susceptibility to nanomaterials and the extent of cytotoxic effects.	[71]
Cytotoxicity Mechanisms	Generation of reactive oxygen species (ROS), inflammation, cell membrane damage.	Oxidation of lipids, proteins, and nucleic acids, activation of inflammatory pathways.	[54]

**Table 6:** Impact of graphene oxide-based polymeric composites on mesenchymal stem cells. Summary of effects on cell behavior and mechanisms.

Authors	Graphene-Based Nanomaterials (Exposure Conditions)	Effect on Mesenchymal Stem Cell	Related Mechanisms	References
Cerverò-Varona; Canciello; Peserico; Haidar Montes et al., 2023	GO [20 µg/mL for 24 h]	Induced and accelerated the EMT	Intracellular activation of TGFβ1-SMAD2/3 signaling pathway	[87]
Xu; Wang; Wu; Fu et al., 2022	GO [0.1, 1, 5, and 10 μg/mL for 24 h]	Induced and accelerated the EMT low-dose GO [0.1, 1 μg/mL]	Activated the Wnt/β-catenin signaling pathway	[47]
Park; Yoon; Lee; Hong et al., 2022	GO [2 μg/mL for 24 h]	Spontaneous surface-induced differentiation of MSCs to SMCs	TGFβ1-induced differentiation	[88]
Halim; Liu; Ariyanti; Ju et al., 2019	GO [0, 0.1, 1, 10, and 100 μg/mL for 0, 24, 48, and 72 h]	Antioxidant response and osteogenic differentiation of bone marrow-derived MSC	Activation and nuclear localization of FoxO1, depending on the JNK activity	[89]
Jagiełło; Sekuła-Stryjewska; Noga; Adamczyk et al., 2019	GO [10–30 µg/cm^2^ and 3–10 µg/cm^2^ for 24, 48, and 72 h] *	Maintenance of the appropriate phenotype of hUC-MSC	Low expression of CD45 and high expression of the CD90 and CD105 antigens	[28]
Zhang; Wei; Li; Li et al., 2020	GO [0.02, 0.1, and 0.5 mg/mL for 24 h]	Increased ROS generation and MMP loss	Upregulation of cleaved caspase-3, LC3-II/I, and beclin-1 and a downregulation of Bcl-2 and caspase-3	[90]

Legend: This table summarizes the impact of graphene oxide-based polymeric composites on mesenchymal stem cells, highlighting the effects on cell behavior and the related mechanisms involved. Abbreviations: GO = graphene oxide; μg/mL = micrograms per milliliter; h = hours; EMT = epithelial–mesenchymal transition; MSC = mesenchymal stem cell; SMC = smooth muscle cell; TGFβ1 = transforming growth factor beta 1; SMAD2/3 = SMAD family member 2 and 3; rGO = reduced graphene oxide; PEG = polyethylene glycol; PEI = polyethylenimine; β-catenin = beta catenin protein; Wnt = Wnt signaling pathway; Cr = creatinine; BUN = blood urea nitrogen; AKI = acute kidney injury; ROS = reactive oxygen species; hUC-MSC = human umbilical cord Wharton’s jelly-derived MSC; MMP = mitochondrial membrane potential; SLA = sandblasted, large grit, acid-etched. * Surface density used on solid substrates. This unit differs from concentrations expressed in µg/mL, which represent the volumetric concentration in liquid media.

**Table 7:** Comparative summary of included studies on graphene oxide-based materials for mesenchymal cell modulation.

Authors	GO or Scaffold Formulation	Model (In Vitro or In Vivo)	Key Findings on MSCs	Noted Limitations	References
Cerverò-Varona; Canciello; Peserico; Haidar Montes et al., 2023	GO-coated glass surfaces	In vitro (amniotic epithelial cells)	Induced EMT (upregulation of Snail, Twist, ZEB, and vimentin; downregulation of CYTO8); activated TGFβ1–SMAD2/3; enhanced migration; increased pro-inflammatory cytokines.	Specific focus on AECs and not directly MSCs; findings in 2D culture.	[87]
Xu; Wang; Wu; Fu et al., 2022	GO (0.1 and 1 μg/mL)	In vitro (MSCs)	Enhanced proliferation and osteogenic differentiation; activated Wnt/β-catenin (increased active β-catenin, *p*-GSK-3β); inhibited chondrogenic and adipogenic differentiation.	Specific GO concentrations; limited scope to osteogenic outcomes in 2D.	[47]
Park; Yoon; Lee; Hong et al., 2022	GO nanosheets (0.1 μg/mL)	In vitro (MSCs)	Promoted MSC proliferation; maintained oxidative balance (upregulated MnSOD, catalase); activated JNK/FoxO1 pathway for osteogenesis.	Specific patterning (100 μm) might limit broader applicability; focus on MSCs.	[89]
Halim; Liu; Ariyanti; Ju et al., 2019	GO-Au nanocomposites	In vitro (MSCs) and in vivo (anti-inflammatory model)	GO-Au(x2) showed highest antioxidant capacity, enhanced proliferation and spreading, and suppressed immune responses (monocyte-macrophage transition, platelet activity).	Limited to low GO doses; primary in vitro assessment.	[90]
Jagiełło; Sekuła-Stryjewska; Noga; Adamczyk et al., 2019	Graphene-based nanostructures	In vivo (acute kidney injury (AKI) model)	Reduced serum Cr and BUN; increased cell proliferation (Ki-67+); reduced apoptosis and necrosis; decreased cyst formation and intratubular debris.	Primarily focused on cytotoxicity and viability and less on lineage-specific differentiation mechanisms.	[28]
Zhang; Wei; Li; Li et al., 2020	Graphene-based nanostructures	In vivo (acute kidney injury (AKI) model)	Enhanced MSC efficacy in kidney regeneration is achieved by improving cell–cell–ECM interactions.	Focus on cytotoxicity and apoptosis mechanisms at high concentrations and not differentiation.	[93]

## Data Availability

The data supporting the findings of this review are publicly available from the MEDLINE-PubMed, EMBASE, and Cochrane Library databases, as described in the Methodology section. No new datasets were generated or analyzed specifically for this study.

## Abbreviations

The following abbreviations are used in this manuscript:

3D	Three-dimensional
AKI	Acute kidney injury
AMSC	Adipose-derived mesenchymal stromal cell
APC	Adenomatous polyposis coli
BUN	Blood urea nitrogen
BMP-2	Bone morphogenetic protein 2
BMP-4	Bone morphogenetic protein 4
BMP-7	Bone morphogenetic protein 7
CK1α	Casein kinase 1 alpha
Cr	Creatinine
ESCs	Embryonic stem cells
MSCs	Mesenchymal stem cells
DMEM	Dulbecco’s Modified Eagle Medium
EGF	Epidermal growth factor
EMT	Epithelial–mesenchymal transition
ROS	Reactive oxygen species
FGF	Fibroblast growth factor
FGF-2	Fibroblast growth factor 2
FoxO1	Forkhead box O1
GO	Graphene oxide
GSK3β	Glycogen synthase kinase 3 beta
hUC-MSC	Human umbilical cord-derived mesenchymal stem cell
IGF-1	Insulin-like growth factor 1
IL-10	Interleukin-10
IL-6	Interleukin-6
JNK	c-Jun N-terminal kinase
ECM	Extracellular matrix
MnSOD	Manganese superoxide dismutase
MMP	Mitochondrial membrane potential
MMPs	Matrix metalloproteinases
MSC	Mesenchymal stem cell
PEG	Polyethylene glycol
PCL	Polycaprolactone
PDGF	Platelet-derived growth factor
PLLA	Poly(L-lactic acid)
PP2A	Protein Phosphatase 2A
SLA	Sandblasted, large-grit, acid-etched
SMC	Smooth muscle cells
TAZ	Transcriptional coactivator with PDZ-binding motif
TCF/LEF	T-cell factor/lymphoid enhancer-binding factor family transcription factor
TGF-β	Transforming growth factor beta
TNF-α	Tumor necrosis factor alpha
VEGF	Vascular endothelial growth factor
Wnt-3a	Wnt family protein, subtype 3a
WNT-4	Wnt family protein, subtype 4
